# Silica-Based Supported Ionic Liquid-like Phases as Heterogeneous Catalysts

**DOI:** 10.3390/molecules27185900

**Published:** 2022-09-11

**Authors:** Anna Wolny, Anna Chrobok

**Affiliations:** Department of Chemical Organic Technology and Petrochemistry, Faculty of Chemistry, Silesian University of Technology, Krzywoustego 4, 44-100 Gliwice, Poland

**Keywords:** ionic liquids, acidic ionic liquids, supported ionic liquid phase, heterogeneous catalysis, silica, immobilization

## Abstract

Supported ionic liquid phases offer several advantages related with catalysis. Immobilization of ionic liquid on the solid support provides catalytic activity or efficient matrix for active phases, as enzymes or metal compounds. Ionic liquid can be physically adsorbed on the carrier (supported ionic liquid phase) or chemically grafted to the material surface (supported ionic liquid-like phase). The use of supported ionic liquid phases improves mass transport, reduces ionic amount in the process and, most importantly, enables effortless catalyst separation and recycling. Moreover, chemical modification of the surface material with ionic liquid prevents its leaching, enhancing length of catalyst life. Silica-based materials have become an effective and powerful matrix for supported ionic liquid-like phase due to its cost-efficiency, presence of hydroxyl groups on the surface enabling its functionalization, and specific material properties, such as the size and shapes of the pores. For these reasons, supported ionic liquid-like phase silica-based materials are successfully used in the organic catalysis.

## 1. Introduction

In recent years, responsible production and consumption has been one of the main topics of interest in both academia and industry. The chemical industry generates large amounts of hazardous waste, along with high energy consumption, use of volatile organic solvents, expensive equipment, and often harsh work conditions [1,2]. Subsequent restrictive regulations concerning health, climate, and environmental protection have forced the chemical industry to improve its existing technologies. The 2030 Agenda for Sustainable Development, adopted by all United Nations Member States in 2015, provides 17 Sustainable Development Goals. New rules for green chemistry can be a useful tool to increase the use of green technologies and achieve sustainable development in the chemical industry [3]. Green catalysis is focused on the minimization or preferably the elimination of waste, relying on the atom economy concept and the search for new effective catalysts while avoiding toxic substances. The newly developed catalysts should be characterized by high activity, selectivity, and stability under the specific process conditions [4]. Meaningful alternatives for conventional hazardous and usually expensive catalysts are enzymes and ionic liquids [5,6].

Ionic liquids (ILs), also known as low-temperature molten salts, are compounds consisting of an organic cation and an organic or inorganic anion. A major advantage of ILs is the possibility of designing their structure by selecting the proper cation and anion while projecting specific properties, meaning they have many applications in the chemical industry [7,8]. Firstly, ILs are significant alternatives for the conventional volatile organic solvents [8]. For example, in the Bayer–Villiger oxidation of ketones in the presence of ILs, lactones and esters are obtained in short reaction times (2–20 h) and in high yields (up to 95%) [9]. ILs can also stabilize enzymes in an active conformation and enhance biocatalytic processes [10]. For example, 1-butyl-3-methylimidazolium bis(trifluoromethylsulfonyl)imide ([bmim][NTf_2_]) was used as solvent in the chemo-enzymatic oxidation of cyclobutanones and cyclohexanones to lactones with high yields (79–95%) in the presence of 30% hydrogen peroxide. In this case, the IL improved the stability of the enzyme under harsh reaction conditions [11]. The ionic nature of ILs also makes them useful as electrolytes for lithium-ion batteries and supercapacitors [12]. Furthermore, ILs are known as extractive solvents for the isolation of high-added value compounds from biomass [13], extractive solvents for analytical chemistry [14], and absorbents for gas capture, e.g., carbon dioxide [15]. ILs can also be employed as catalysts or solvent and catalyst at the same time in many reactions, e.g., Diels–Alder cycloaddition, alkylation, and acylation, as well as various types of condensations, oxidation, esterification, and transesterification reactions [16,17].

**Figure 1:**
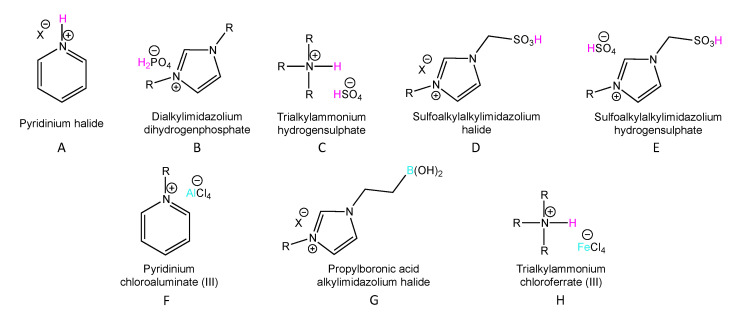
Examples of the structures of some acidic ionic liquids.

One significant group of ILs that are used as catalysts are acidic ionic liquids (AILs). AILs can be classified according to the nature of the acidic site on the Brønsted and Lewis acid types. It is possible to introduce more than one acidic function to the structure of AILs and design ILs by the combination of Brønsted and Lewis acidic types. Brønsted acidity can be introduced to ionic liquids (BAILs) as either: an acidic hydrogen in the cation (A), an anion (B) or both (C), an acidic hydrogen located in the functional group (D) or an acidic hydrogen located in the functional group and in cation/anion (E). Lewis acidic ionic liquids (LAILs) are mainly based on halometallate anions (F) and boric atom in the cation (G) (Figure 1). The formation of dual Brønsted–Lewis AILs is also presented in Figure 1 (H) [18,19,20].

The most common group of BAILs are ILs with an acidic hydrogen located at the cation (A), which are also called protic acidic ionic liquids. Cations widely used for the synthesis of this type of BAIL are: 1-alkylimidazolium, 1-alkyl-2-alkylimidazolium, primary/secondary/tertiary ammonium, pyridinium, pyrolidonium, and 1,1,3,3-tetramethylguanidininium [19]. A functional group with an acidic hydrogen (e.g., -SO_3_H, -CO_2_H) can also be attached to the cation to obtain a BAIL [20]. An acidic site in the BAIL’s anion is formed using polybasic acids such as H_2_SO_4_, H_3_PO_4_, maleic, and fumaric acids creating dialkylimidazolium, hydrogensulfate, or dihydrogenphosphate ILs [20,21,22]. A growing interest in green chemistry has also led to the discovery of bio-BAILs based on amino acids introduced into the structure of the cation or anion, e.g., alanine, glycine, serine, proline, and valine [23]. BAILs have been implemented in many organic reactions. For example, imidazolium-based ionic liquids functionalized with a sulfonic group were successfully employed for the hydration of alkynes under mild conditions to give ketones in high yields [24]. Some dicationic ionic liquids based on a diammonium cation and hydrogensulfate anion as environmentally benign BAILs were used for biodiesel synthesis, which was obtained with high yields and reused without significant loss of activity [25]. Imidazolium based hydrogensulfate ILs were also determined to be very efficient catalysts in the synthesis of cyclic carbonates from carbon dioxide and epoxides. High yields of cyclic carbonates (69–99%) were achieved using these ILs, which can be recycled without any loss of activity [21]. In another example for the dehydration of glycerol to acrolein using the BAIL 1-butyl-3-metylimidazolium dihydrogen phosphate, which was conducted in the liquid phase, full conversion of glycerol was achieved [22].

For the Lewis AILs, metals such as Al, Ga, Zn, Fe, In, and Sn in the form of chloride or triflate salts are used to create LAILs via complexation of the neutral IL and the metal salt in various molar ratios [18,26]. Lewis acidic cations can be formed in two ways: via a tricoordinate borenium center as a cation, or via solvation of metal cation, e.g., Li^+^ as [Li(glyme)][NTf_2_]/[OTf] [18]. LAILs, as well as BAILs, are readily used in organic synthesis. Water tolerant trifloaluminate ILs, synthesized from 1-alkyl-3-methylimidazolium triflates, were employed as catalysts in the cycloaddition of 2,4-dimethylphenol and isoprene to obtain a chromane. Use of the catalysts provided full conversion and high selectivity (80%) under mild reaction conditions [26]. In another example, chlorogallate(III) ILs were applied in a Bayer–Villiger oxidation of cyclic ketones to lactones. High yields (99%) in short reaction times under mild reaction conditions were also achieved [27]. Borenium LAILs used in a Diels–Alder reaction ensured good yields (90–94%) and selectivities of various dienes and dienophiles [28]. All such AILs have many applications as homogeneous catalysts [18,19], however, reducing costs and waste led to the use of heterogeneous catalysis.

**Figure 2:**
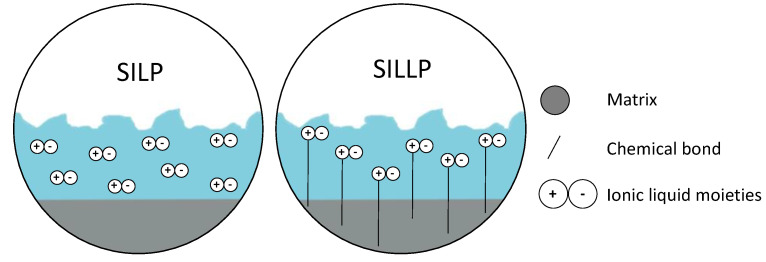
Supported ionic liquid phase (SILP) and supported ionic liquid-like phase (SILLP).

Immobilization of ILs on a solid insoluble support can be performed via physical adsorption, known as supported ionic liquid phase (SILP), or via chemical bonding into the matrix, known as supported ionic liquid-like phase (SILLP) [29]. A visual representation of each can be seen in Figure 2. The IL creates a thin layer of liquid on the carrier, which decreases the amount of IL compared to the reaction in the bulk. This improves mass transfer to the catalytic centers on the fluid-fluid phase boundary and facilitates separation of the catalyst from the reaction mixture. Moreover, a heterogeneous SILP or SILLP catalyst can be successfully employed in both batch and flow processes, including fixed-bed or fluidized-bed reactors. Such applications are described later in this paper.

In this paper, achievements on the SILLP silica-based materials and their use in the organic synthesis are described. Previously, Mehnert [30] outlined the first contribution of SILPs in catalysis. Then, Sokolova et al. [31] reviewed flow processes based on catalysts immobilized on monolithic SILLPs. Next, Skoda-Földes [32] summarized the use of supported AILs in the organic synthesis, and Hartmann et al. [33] characterized inorganic materials for SILLP synthesis and briefly described their input to catalysis. After that, Amarasekara [20] characterized AILs and described applications of acidic ionic liquids as SILP/SILLP, and Gruttadauria et al. described covalently-supported ionic liquid phases (SILLP) as matrices and catalysts [34], while Alinezhad et al. pointed out BAILs as SILLP in organic catalysis [35]. Then, Swadźba-Kwaśny et al. [18] briefly mentioned the applications of Lewis ILs immobilized into a solid matrix, and Leitner et al. [36] described SILP and SILLP based on nanoparticles and their applications in organic catalysis. Additionally, Vekariya [16] mentioned SILPs in the review of ILs in organic transformations. Haumann et al. [37] then presented 15 years of using SILP/SILLP catalysts in hydroformylation reactions, both in the liquid and gas phase, and Freire et al. [38] described the immobilization of ionic liquids, types of materials, and their applications. Maciejewski et al. described participation of ILs in heterogeneous catalysis, including supported IL phase catalysts (SILPC), solid catalysts with ILs (SCILL), and supported ionic liquid catalysis (SILC) techniques, as well as porous ionic liquids [39]. Moreover, Lozano et al. [40] presented applications of SILP and SILLP as supports for enzyme immobilization in organic synthesis, and Chrobok et al. [41] described SILP/SILLP biocatalysts based on nanoparticles and their applications for biocatalysis. The aim of this work is to complete the time gap and collect silica-based SILLP applications in catalysis to improve selection of the best systems for organic synthesis.

## 2. Immobilization of Ionic Liquids on Silica-Based Materials

The immobilization of ILs on the solid supports enables the issues related with the bulk IL systems to be overcome, such as high viscosity, mass transfer problems, IL high-cost separation, purification, regeneration, and recycling. A reduced amount of immobilized IL creates a thin layer on the matrix which, in turn, reduces costs. The possibility of creating numerous structures of ILs caused various SILPs to be designed, generating wide application potentials. Different types of materials such as silica, alumina, zeolites, polymers (e.g., polystyrene-based materials), and carbon materials (e.g., single-walled carbon nanotubes (SWCNTs), multi-walled carbon nanotubes (MWCNTs), and activated carbon) were used for such SILPs [37,41]. The most commonly used matrices are silica-based materials (e.g., silica gel, SBA-15, MCM-41 types), which are characterized by their low cost, large surface area, ordered porosity, well-defined pore geometry, and mechanical and thermal stability (except for MCM-41 type). Moreover, magnetic properties can be incorporated by coating Fe_3_O_4_ nanoparticles with silica, obtaining a hybrid that is even easier to separate from the reaction mixture using a magnetic field. The most important feature of silica-based materials is the presence of silanol groups (-Si-OH) on the surface, which determines the method of IL immobilization, particularly via covalent bonding (SILLP).

**Figure 3:**
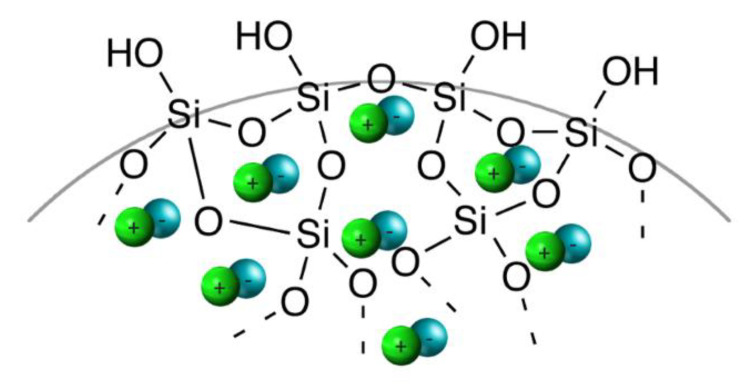
Ionic liquids moieties entrapped in the silica pores.

Physisorption is a simple method for IL immobilization that can be performed through the impregnation and adsorption from IL solution and the sol-gel procedure. The impregnation method relies on mixing the IL solution and support together before removing the solvent under vacuum conditions. The adsorption from the IL solution is accomplished by filtration, washing (to remove any excess IL), and drying under vacuum conditions. The sol-gel procedure consists of hydrolysis and polycondensation reactions of tetraethoxyorthosilicate (TEOS) in the presence of the IL, which can be described by the entrapment of the IL in the silica pores (Figure 3). The main strength of the sol-gel technique is that there is control of the molecule’s growth [42]. The interactions between the IL and the silanol groups on the silica surface are based on hydrogen bonding. However, van der Waals and electrostatic interactions, as well as π-π-stacking (in the case of aromatic cation) between the IL moieties also occurs, increasing the stabilization of the SILP structure [43]. The H-bonds between -Si-OH and the IL can be confirmed via FTIR analysis, where the intensity of the characteristic peak at 952 cm^−1^ (assigned to -Si-OH) decreases if IL is present on the silica surface [44].

SILPs are commonly used for both chemical and biochemical processes. Entrapped triethylammonium propanesulfonate bis(trifluoromethanesulfonyl)imide [TEAPS][NTf_2_] in the silica structure has been used for dehydration of *rac*-1-phenyl ethanol with high selectivity to styrene and recyclability (for at least 6 runs) [45]. Another BAILs, 1-methyl imidazolium hydrogen sulphate ([HMIM]HSO_4_) and 1-methyl benzimidazolium hydrogen sulphate ([HMBIM]HSO_4_), immobilized on silica, was applied in the isomerization of *n*-heptane and *n*-octane. The acidic SILPs showed good thermal stability high isomerization yields, were easy recyclable and environmentally friendly [46]. Then, 1-butyl-3-methylimidazolium acidic ILs with Rh-complex were immobilized on partly dehydroxylated silica surface, which created a highly active Rh/SILP catalyst dedicated for continuous hydroformylation of propene. High thermal stability, selectivity to *n*-butanal (over 95%), and TOF (turnover frequency) were observed using syn-gas and syn-gas with CO_2_ addition [47]. For the hydrosilylation reaction, rhodium complexes immobilized in four various phosphonium based ILs anchored on silica support were applied. The amount of catalyst was reduced compared to biphasic reactions by a factor of 1000, the reaction times were shortened, and easy recycling of the Rh complexes were demonstrated [48]. The advantage of SILP catalysts in biocatalysis has also been shown. Lipase B from *Candida antarctica* (CALB) was immobilized on a SILP based on an imidazolium cation and a bis(trifluoromethanesulfonyl)imide anion used for a continuous kinetic resolution of 1-phenylethanol under supercritical CO_2_ conditions. High enzyme activity, enantioselectivity (>99.9%), and stability (16 cycles) was achieved [49]. SILP catalysts have many advantages, such as easy and cost-efficient synthesis, where an IL multilayer on the support maintains the IL bulk properties, as well as the possibility to tailor the structure of the ILs that can be immobilized. It is worth noting that the main disadvantage is the detachment or leaching of the IL from the matrix, which is related to weak interactions between the IL and the carrier.

**Figure 4:**
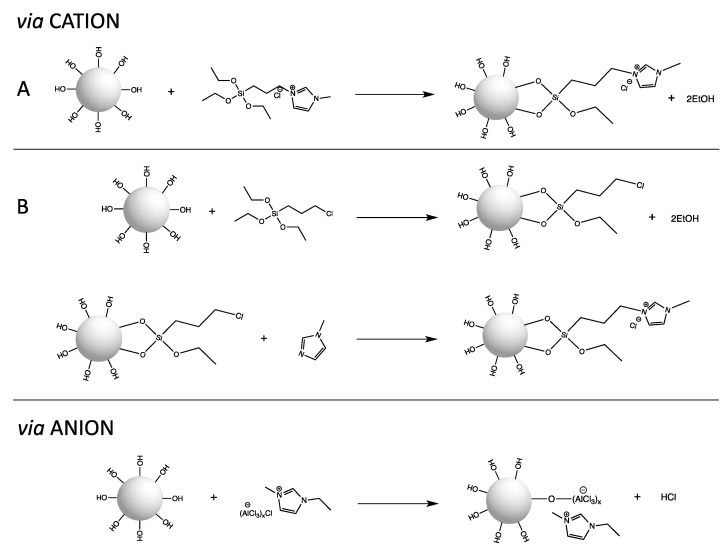
Covalent immobilization of ionic liquid on the silica surface via cation and via anion. Anchoring the IL into support can be obtained by direct immobilization of IL (**A**) or by building the IL structure on the support (**B**).

Covalent bonding of the IL on the surface of the support prevents its leaching and detachment. ILs immobilized as SILLPs usually create a monolayer, thus the bulk properties are lost. Methods for the preparation of SILLP silica-based materials include chemical reactions between an IL or IL precursor and hydroxyl groups present on the silica surface, or the sol-gel technique. ILs can be attached to -Si-OH group via the cation or the anion (Figure 4). Anchoring the IL into support can be obtained by direct immobilization of IL (Figure 4A) or by building the IL structure on the support (Figure 4B).

**Figure 5:**
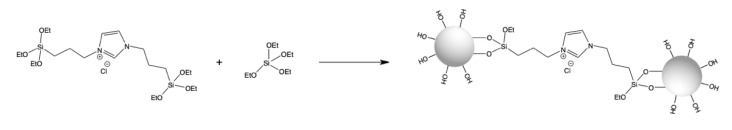
Covalently immobilized ionic liquid via the sol-gel method.

Typical immobilization of the IL via the cation is performed using the siliceous precursor 3-(chloropropyl)triethoxysilane, however, other precursors can also be used, e.g., 3-mercaptopropyl-trimethoxysilane [32]. As mentioned before, prepared in advance, an IL modified with ethoxysilane groups can be directly grafted to hydroxyl groups or precursors, and can be firstly anchored and be the subject of subsequent quaternization. Immobilization of IL via the cation to the siliceous surface can be confirmed by ^29^Si MAS NMR. Peaks at −91 ppm and −101 ppm assigned to (SiO)_2_Si-(OH)_2_ and (SiO)_3_Si-OH groups, respectively, disappear, thus exposing the (SiO)_4_Si signal. Signals at −54 ppm and −66 ppm assigned to -Si-O-SiR-(OEt)_2_ and (Si-O)_2_-SiR-OEt, respectively, are in turn revealed [29]. If necessary, an anion exchange can be performed after the IL immobilization. Immobilization of the IL via the anion is usually observed mainly for chlorometallate ILs during the wet impregnation method where -Si-O-M bonds are obtained. For example, ^27^Al MAS NMR spectra shows signals at 102 ppm attributed to [Al_2_Cl_7_]^−^ and allows the control of the presence of AlCl_3_ on the silica surface (1.2 ppm), which can be removed though the Soxhlet extraction [29,32]. The sol-gel method is also often used for SILLP preparation. This technique consists of polycondensation of alkoxysilane-functionalized ILs with tetralkoxysilanes, e.g., TEOS (Figure 5), and allows the control of material mesoporous character from the proper silica source/IL ratio. Besides MAS NMR spectroscopy, chemical immobilization of IL to the silica surface can be proved using FT-IR, XRD, and TEM methods.

## 3. Silica-Based Supported Ionic Liquid-like Phases in Organic Catalysis

### 3.1. Lewis Type SILLPs

**Figure 6:**
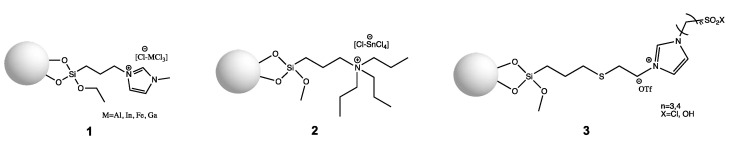
Structures of Lewis type silica-based SILLP materials.

Lewis type SILLPs based on silica materials are normally synthesized via the cation method using 3-(chloropropyl)triethoxysilane, 3-(chloropropyl)trimethoxysilane, or 3-mercaptopropyltrimethoxysilane precursors, where the structures shown in Figure 6 are obtained. As can be seen in Figure 6, the material can be characterised by Lewis acidity with the Lewis center located on the alkyl chain modified with -SO_2_Cl or -SO_2_OH groups, or the Lewis acidity can be found in the metal halide based anion created in the complexation reaction. Table 1 presents applications of Lewis type silica based SILLP materials as catalysts in organic synthesis.

**Figure 7:**
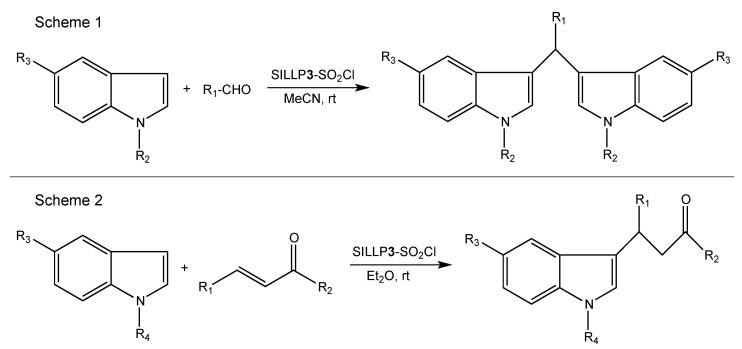
Applications of Lewis type SILLP material **3** in the synthesis of bis(indolyl)methanes (Scheme 1) and addition of indole to vinyl ketones (Scheme 2).

The first report on Lewis type SILLPs appeared in 2000. The presented investigations included two different immobilization methods of chloroaluminate imidazolium ILs on amorphous silica and MCM-41 supports. One of the possible SILLP synthesis routes was immobilization via the anion (Figure 4), with the second one being via the cation (**1**, Figure 6), where aluminium chloride was introduced to the IL structure in the complexation reaction. If the molar ratio of the metal halide component in the IL is more than 0.5, oligonuclear ([Al_2_Cl_7_]^−^) anions are formed. The obtained heterogeneous catalysts were tested on the Friedel–Crafts alkylation, which resulted in high conversion (>90%) and selectivity (>90%) of the main product using catalyst **1** in Figure 6. In comparison, the reaction catalysed by immobilised AlCl_3_ on the silica surface yielded only 15.7% of the main product. The better activity shown by the MCM-41 based SILLP is due to higher surface area and IL loading. Furthermore, leaching of the active phase occurred for the SILLP catalyst prepared by anion immobilization, partly due to unbonded IL moieties on the silica surface [29]. In the next report, chloroaluminate SILLP catalysts prepared via anion, cation, and sol-gel methods were used in the Friedel–Crafts alkylation of benzene with different olefins, as well as in acylation reaction. Again, leaching of the IL occurred in the SILLP prepared via anion complexation. The best activity was shown by the SILLP catalyst, where IL was grafted via the cation—almost full conversion and very high selectivity of the monoalkylated product were achieved, even at 20 °C. The lower activity of other SILLPs was most likely the result of only partly bonded acidic anions on the silica surface, which was confirmed via ^29^Si MAS NMR analysis [50]. A tetrapropylammonium based chlorostannate (IV) IL was grafted to the silica surface via the cation (**2**, Figure 6), and used in the condensation of isobutene and formaldehyde to 3-methylbut-3-en-1-ol. Comparison of silica and MCM-41 materials resulted in better activity of the MCM-41 based SILLP catalyst in the tested reaction (α = 76%, S = 94%, Y = 71.4%, TON = 2.63⋅10^−3^ s^−1^). Well-ordered and regular hexagonal pores in the MCM-41 material created micro-reactors that enhanced the SILLP catalyst activity. It is worth mentioning that the obtained heterogeneous catalyst was recyclable, and that the active phase can be used as a catalyst in the homogeneous phase as well [51]. The next report described applications of triflate Lewis type SILLP materials (**3**, Figure 6) in the synthesis of bis(indolyl)methanes [52] (Scheme 1, Figure 7), esterification of acetic or decanoic acid with various alcohols [53], nitration of aromatic compounds [53], and the addition of indole to vinyl ketones [54] (Scheme 2, Figure 7). Covalent bonding between the IL and hydroxyl groups on the silica surface was created in a radical chain transfer reaction of a 1-allylimidazolium based IL on silica gel modified with 3- mercaptopropyltrimethoxysilane. The obtained materials exhibited excellent yields, conversions, and reusability in all presented reactions. It should be pointed out that the replacement of chloroaluminate anion to triflate, and creation of Lewis centre in the cation, makes SILLP materials more resistant to water.

**Figure 8:**
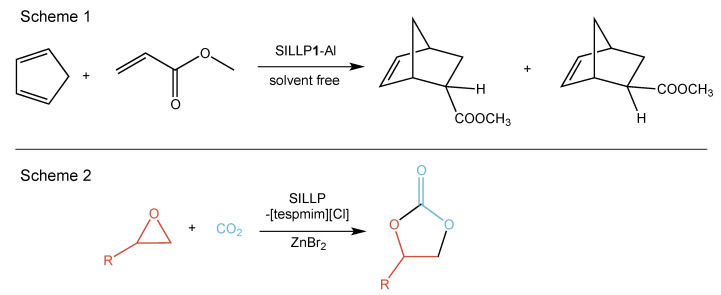
Diels–Alder cycloaddition (Scheme 1) and CO_2_ cycloaddition for cyclic carbonates synthesis (Scheme 2) with SILP catalysts.

Further reports have presented a chloroaluminate imidazolium-based IL grafted to MCM-41 (**1**, Figure 6). High conversion (97%) and selectivity to isooctane (59.7%) in gasoline production were obtained, and the SILLP catalyst showed better activity than the IL immobilized via the anion, zeolite H-Beta, and Nafion/Silica Composite SAC 13 [55]. The same IL was immobilized on silica, MCM-41, SBA-15, active carbon, and glass materials, and the activity of the prepared SILLP was examined in the continuous gas phase trimerization of isobutene using a fixed-bed reactor under atmospheric pressure. Only silica-based SILLP catalysts enabled the trimerization reaction to occur due to synergic interactions between the IL anion and the silanol groups. In other cases, the alkylation reaction was observed. For the trimerization reaction, the MCM-41 based SILLP (α = 91.4%, S_C12_ = 79.4%) turned out to be the most active, owing to its regular hexagonal array channels that behave similar to micro-reactors, increasing the catalytic activity [56]. Apart from Al (III) and Sn (IV), other metals such as Fe, In, and Ga were used for silica-based SILLP synthesis. Chloroferrate (III) imidazolium-based IL moieties were grafted for the siliceous support of MCM-41 after complexation with FeCl_3_ (**1**, Figure 6). The SILLP catalyst showed high efficiency and long reusability (10 cycles) in the Friedel–Crafts reaction between benzene and benzyl chloride [57]. The chloroindate (III) imidazolium-based IL was anchored to SBA-15 silica material (**1**, Figure 6), which exhibits ordered hexagonal structure, however less so than MCM-41 material. The obtained SILLP catalyst was used in the Friedel–Crafts reaction between benzene and benzyl chloride, gaining 100% conversion and 100% selectivity over 6 reaction cycles. Introducing the IL to the catalyst structure prevents InCl_3_ from leaching [58]. A chlorogallate (III) imidazolium-based IL was covalently tethered to a multimodal silica porous silica support (**1**, Figure 6) and applied to Diels–Alder cycloaddition reactions for the synthesis of intermediates for pharmacologically active ingredients, agrochemicals, flavors, and fragrances (Scheme 1, Figure 8). The synthesized materials demonstrated a hierarchical pore structure and contained micro-, meso-, and macropores, which results in easy mass transport to and from active sites. The SILLP chlorogallate (III) catalyst showed great conversions and endo/exo selectivities in short reaction times, which is superior to other results presented in the literature. Moreover, the catalyst could be recycled five times without significant loss of activity [59].

Other reports concern the application of ILs as co-catalysts for CO_2_ cycloaddition for cyclic carbonates synthesis in the presence of a Zn Lewis centre (Scheme 2, Figure 8). It is postulated that the Zn Lewis site coordinates with the oxygen atom of epoxides, and a nucleophilic attack of the halide anion on the less sterically anion carbon atom of epoxide occurs. The first approach includes [tespmim][Cl] anchored to various silica materials such as macro/mesoporous silica, MCM-41, MSU-F (cellular foam), and MSU-H (large pore 2D hexagonal). The better results in the reaction of CO_2_ with styrene oxide in the presence of ZnBr_2_ were achieved for the silica SILLP, and the worst occurred for the MCM-41 SILLP. In this case, catalytical activity depends on pore size and not on surface area. The synthesised catalyst could be recycled four times without any loss of activity [60]. In other reports, Zn atoms and [tespmim][Cl] were grafted to the silica surface. Various materials such as MCM-41 (regular, long, hexagonal channels), MSN (nanosphere morphology, order mesopores, mainly inside pores), and BMMs (mesoporous structure, a large number accumulated inside and outside of the pores) were applied. The catalytic activity of the SILLP catalyst was examined in reaction of CO_2_ with propylene oxide, where the best performance was exhibited by SILLPs with shorter and regular pore channels [61].

### 3.2. Brønsted-Type SILLPs

**Figure 9:**
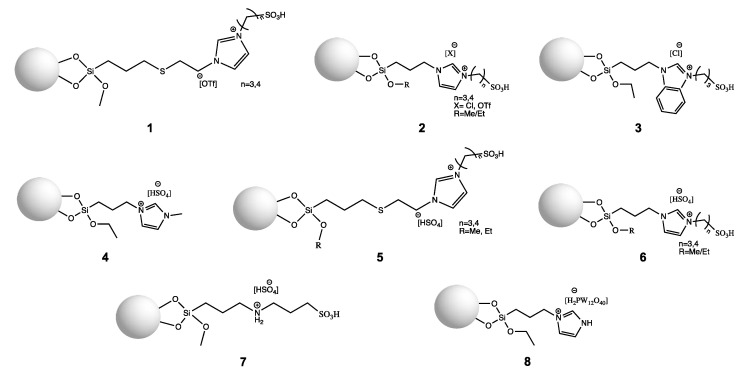
Structures of Brønsted-type silica-based SILLP materials.

Brønsted-type SILLPS based on silica materials are synthesized via the cation method. Brønsted IL moieties are grafted to the silica surface through precursors such as 3-(chloropropyl)triethoxysilane, 3-(chloropropyl)trimethoxysilane, 3-mercaptopropyltrimethoxysilane, or (3-aminopropyl)-trimethoxysilane, creating the structures presented in Figure 9. The Brønsted acidic center located in the anion that is most often used is [HSO_4_], whereas the cation Brønsted site can be found in the alkyl chain modified with an -SO_3_H group through the reaction between, for example, 1,3-propanesultone and vinylimidazole. Some applications of Brønsted-type silica-based SILLP materials as catalysts in organic synthesis are presented in Table 2.

**Figure 10:**
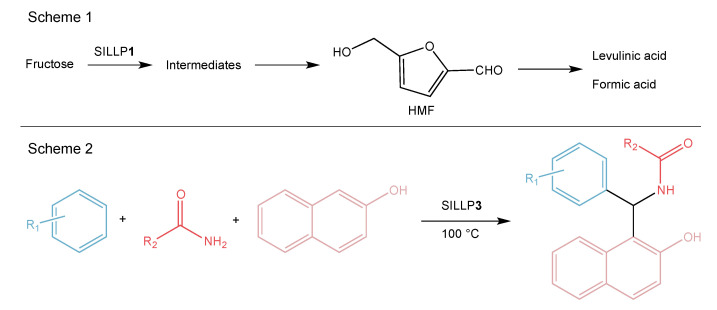
Brønsted-type SILLP based on silica material in the dehydration of fructose (Scheme 1) and synthesis of 1-amidoalkyl naphthols (Scheme 2).

Brønsted acidic vinylimidazolium-based IL moieties modified with -SO_3_H groups were grafted to a sulfhydryl group-modified silica surface through free radical addition obtaining SILLP catalyst **1** (Figure 9). The prepared material was used in the esterification of oleic acid with methanol and the transesterification of glycerol trioleate with methanol. It was reported that the loading density of the IL influenced both reactions. Increasing the loading of the IL on the support induced the conversion of oleic acid. However, for the conversion of glycerol trioleate, the opposite effect is observed. This is due to the size of glycerol trioleate molecules and the decreasing pore size of the SILLP. The SILLP catalyst could be used in the esterification for three cycles, after which the catalytic activity dropped and the hydrolysis or alcoholysis of the -Si-O-Si- bonds occurred [62]. In the following report, the structure of a Brønsted imidazolium IL modified with -SO_3_H groups was produced in three stages: first, 3-(chloropropyl)trimethoxysilane was anchored to the silica surface. Next, imidazole moieties were introduced to the structure before 1,4-butanesultone was used to modify the imidazole ring with a -C_4_H_9_SO_3_H group (**2**, Figure 9). The catalytic activity of the obtained SILLP material was tested in a cellulose hydrolysis and yielded a 48.1% reduction of sugar and 21.9% of glucose. In this case, the catalyst maintained activity for three cycles. Due to the use of the SILLP catalyst, the total yields in the reduction of sugar and glucose were higher than using SO_3_HC_3_H_7_-SiO_2_ or SO_3_H-SiO_2_, keeping the same -SO_3_H group loading. This effect results from the interaction between the imidazolium IL and the hydroxyl groups in cellulose [63]. The SILLP catalyst **1** (Figure 9) was also used in the dehydration of fructose to 5-hydroxymethylfurfural (HMF) with 100% conversion and 70.1% yield of the main product (Scheme 1, Figure 10). In this reaction, the catalyst was reused without significant loss of activity for 7 cycles. Simultaneously, the same SILLP material with a Lewis -SO_2_Cl center was tested for this reaction. However, the catalyst exhibited inferior efficiency compared with the Brønsted-type SILLP [64]. The next report described the functionalization of bifunctional periodic mesoporous organosilica with IL and -SO_3_H groups, as well as its application in a Biginelli condensation reaction for the synthesis of pharmacological and biological activities compounds. The novel material assured high yields of various products (Table 2) and could be recycled over 10 times without any decrease in efficiency [65]. The same catalyst was also used in the esterification of acetic acid with various alcohols and, again, high yields of the main products were reached and the SILLP material could be reused several times [66]. Another report mentioned a triflate imidazolium-based IL with a -SO_3_H group anchored to the silica, MCM-41, and SBA-15 materials (**2**, Figure 9). Activity tests were performed for the self-condensation of pentanal to 2-propyl-2-heptenal, where the best results were achieved for the silica based SILLP (69.4% yield, 89.6% selectivity), which was due to the highest IL loading on the surface [67]. A benzimidazolium IL with a -SO_3_H group in alkyl chain was grafted to silica surface (**3**, Figure 9) in stages (which were described above). The SILLP catalyst was employed in the transesterification of non-edible oils with high free fatty acids, as well as for the synthesis of 1-amidoalkyl naphthols from 2-naphthol, amides, and aldehydes (Scheme 2, Figure 10). This eco-friendly and efficient catalyst for transesterification provided 95% yield of fatty acid methyl esters and catalytic stability over 5 runs [68]. Moreover, the SILLP material in the synthesis of 1-amidoalkyl naphthols exhibited high yield of the obtained products (Table 2), high product quality, short reaction times, and reusability for five reaction cycles, which makes the catalyst very useful for industrial practices [69].

**Figure 11:**
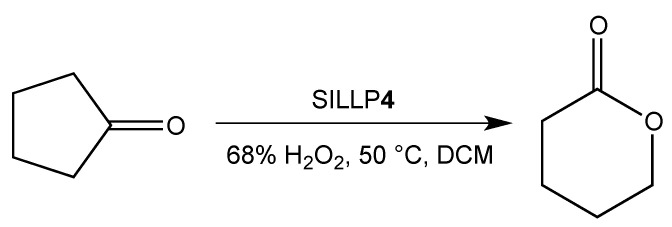
Brønsted-type SILLP based on silica material in Bayer–Villiger oxidation.

**Figure 12:**
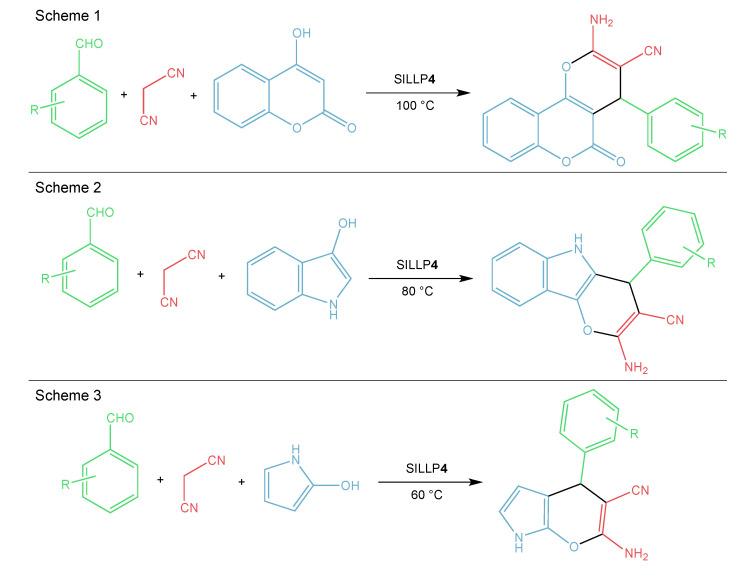
Brønsted-type SILLP based on silica material in the synthesis of 3,4 -dihydropyrano[c]chromenes (Scheme 1), pyrano[3,2-b]indoles (Scheme 2), and pyrano[2,3-b]pyrroles (Scheme 3).

Further reports concern the application of covalently immobilized imidazolium-based ILs with a hydrogensulfate anion on silica materials (**4**, Figure 9). The Bayer–Villiger oxidation of cyclic ketones to lactones (Figure 11) is one example of numerous reactions catalyzed by SILLP **4** (Figure 9). For that purpose, a silica material with the extensive system of meso- and macropores was used. Here, the catalyst showed great activity, which resulted in high conversions of ketones and yields of lactones (60–91%), short reaction times, and good reusability (three cycles) [70]. The same SILLP catalyst was used in the esterification of acetic acid with butanol with a 99.4% conversion, and a reusability of six catalytic cycles with a slight decrease of conversion were observed [71]. The synthesis of 1-(benzothiazolylamino)phenylmethyl-2-naphthols catalyzed by SILLP **4** (Figure 9) was also reported. In this case, IL was anchored to rice husk ash, which is a natural source of amorphous silica. High yields for various aldehydes (90–93%) and high TOF (92 h^−1^) were achieved (Table 2) in very short reaction times. Furthermore, the catalyst could be reused six times without activity loss [72]. The same catalytic system was examined for the formylation of amines. Again, the catalyst proved to be simple, stable, and efficient, since high yields (93–97%), TOF (465–7275 h^−1^) and reusability over 10 cycles were reached [73]. In another report, an IL containing a hydrogensulfate anion was immobilized on nanoporous silica SBA-15 and used in the synthesis of hexahydroquinolines via the Knoevenagel–Michael cyclization as an alternative to conventional catalysts. Excellent yields (90–93%), short reaction times, aqueous conditions, and reusability (seven runs) made the process more environmentally friendly [74]. The synthesis of 3,4-dihydropyrano[c]chromenes (Scheme 1, Figure 12) and pyrano[2,3-c]pyrazoles were also proceeded in the presence of SILLP **4** (Figure 9). Various ILs with anions, such as [HSO_4_]^−^, [H_2_PO_4_]^−^, [Br]^−^, and [OTf]^−^ were tested, with the best results gained for the hydrogensulfate anion. The catalyst exhibited very good yields (89–95%) and reusability (five cycles) [75]. The use of SILLP **4** (Figure 9) was also successful for the synthesis of pyrano[3,2-b]indoles (Scheme 2, Figure 12) [76], pyrano[2,3-b]pyrroles (Scheme 3, Figure 12) [77], benzo[f]chromenes [78], 2,9-dihydro-9-methyl-2-oxo-4-aryl-1H-pyrido[2, 3-b]indole-3-carbonitriles [79], acenaphtho[1,2b]pyrroles [80], and 5-amino-7-aryl-6-cyano-4H-pyrano[3,2-b]pyrroles [81]. As shown in Table 2, satisfying yields for different aldehydes, arenes, and components were achieved, which indicates the versatility of SILLP 4 (Figure 9) catalyst, as well as the developed methods. Moreover, the catalyst could be reused several times [76,77,78,79,80,81]. The dihydrogenphosphotungstate anion ([H_2_PW_12_O_40_]^−^) was reported in an SBA-15 based SILLP, exhibiting well-ordered, mesoporous specific high surface area. This novel catalyst found application in the oxidation of dibenzothiophene, 4,6-dimethylbenzothiophene, and benzothiophene for fuel desulfurization. This SILLP showed excellent efficiency, with 100% conversion of dibenzothiophene and 4,6-dimethylbenzothiophene, which means the total ability of removal of toxic compounds from the fuel. Furthermore, the catalyst could be successfully reused four times [82].

**Figure 13:**
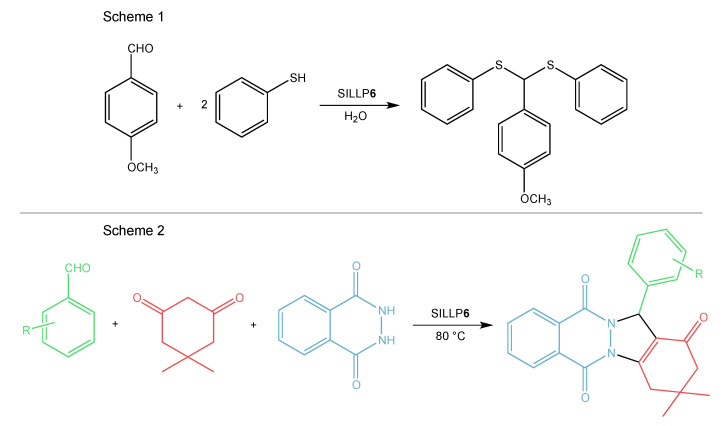
Brønsted-type SILLP based on silica material in the synthesis of thioacetalization of (Scheme 1)), 2H-indazolo[1,2-b]phthalazine-triones (Scheme 2).

Dual Brønsted acidic ILs immobilized on silica materials are another group of Brønsted-type SILLPs. In this case, the Brønsted centers are located both in the cation and anion, like the -SO_3_H groups grafted to the alkyl chain in the cation and like the [HSO_4_]^−^ in the anion (**5**, **6**, Figure 9). This kind of catalyst is quite often used according to the literature. One use is the esterification of acetic acid and n-butanol. SILLP **5** (Figure 9) material caused 99.5% yield of n-butyl acetate, where it could be recycled eight times with only a slight decrease in conversion to 90.1% [83]. The next report presents 3-sulfopropyl-1-(3-propyltrimethoxysilane)imidazolium hydrogensulfate IL anchored to silica gel forming SILLP **6** (Figure 9) in the synthesis of amidoalkyl naphthols by the multicomponent condensation. High yields and TOFs (Table 2) were obtained, and the catalyst kept activity for seven cycles without significant loss [84]. SILLP **6** (Figure 9) was also used as a catalyst in the thioacetalization of carbonyl compounds, providing high yields (85–96%). The reaction between 4-methoxybenzaldehyde with thiophenol (Scheme 1, Figure 13) was characterized by 96% yield, mild reaction conditions, and short reaction times, with the catalyst efficiently being recycled six times [85]. Furthermore, the same catalyst was employed in acetalization of benzaldehyde or furfural with various diols. High catalytic activity (yields 85–96%) for 10 reaction runs was reached for the synthesis of benzaldehyde ethanediol acetal [86]. Again, SILLP **6** (Figure 9) was used as a catalyst in the synthesis of 2H-indazolo[1,2-b]phthalazine-triones (Scheme 2, Figure 13) [87] and polyoxymethylene dimethyl ethers [88]. In the first case, nano-silica formed a matrix for IL immobilization. The synthesized material showed high catalytic activity, gaining 81–96% yield of indazolophthalazine-triones and bisindazolophthalazine-triones, while maintaining activity over seven reaction cycles [87]. Various types of such silica gels used in SILLP synthesis are described widely throughout the literature. In order to reduce the ratio of the catalyst in the reactants, the SILLP with the highest surface area and IL loading was selected as the catalyst. This resulted in a 52% trioxane conversion and 92% polyoxymethylene dimethyl ethers selectivity [88]. Next, SILLP **6** (Figure 9) was also employed in a lignin depolymerization. This highly thermally stable catalyst allowed a 90% yield of tetrahydrofuran soluble products to be obtained in 1 h at 200 °C [89]. It was also found that SILLP **5** (Figure 9) catalyzed the esterification of acetic acid and n-butanol. The catalyst provided a 99.2% yield and 100% selectivity and was active for seven cycles. Moreover, high yields for reactions with various alcohols were achieved (Table 2) [90]. The novel silica-based SILLP **7** (Figure 9) material was also synthesized from the (3-aminopropyl)-trimethoxysilane precursor. The prepared catalyst was used in the acetalization of benzaldehyde with 1,2-ethanedioland and biodiesel synthesis. In both cases, the SILLP exhibited high catalytic activity, assuring 99% yields of the main product and reusability over six cycles. In comparison with conventional biodiesel synthesis, SILLP catalysts are a very promising alternative [91]. SILLP **8** (Figure 9) with Brønsted acidic sites introduced with an imidazolium cation and dihydrogenphosphotungstate anion was tested in the oxidation of alkenes. The catalyst proved to be efficient in this reaction, providing high selectivities, conversions, and TOF (Table 2) [92]. The dihydrogenphosphotungstate anion was involved in the synthesis of SILLP **5** (Figure 9) instead of the hydrogensulfate anion. The SBA-15 SILLP catalyst was applied in the biodiesel synthesis from palmitic acid. In comparison to other anions such as hydrogensulfate and triflate, the dihydrogenphosphotungstate anion performed the highest IL loading and catalytic activity, giving an 88.1% yield and reusability of over five times [93].

### 3.3. Fe_3_O_4_-Silica Hybrid Based SILLPs

**Figure 14:**
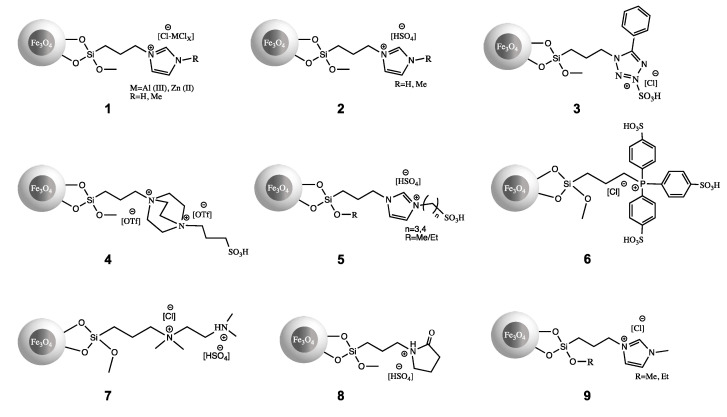
Structures of silica-Fe_3_O_4_-based SILLPs.

Immobilization of an ionic liquid on a solid matrix provides easy catalyst separation from the reaction mixture, as well as its recycling. Doping silica materials with Fe_3_O_4_ offers new features, such as magnetic properties, for example. A silica-Fe_3_O_4_ hybrid could be even faster and more easily separated from the reaction mixture using an external magnetic field, making it an attractive support. Table 3 shows applications of silica-Fe_3_O_4_-based SILLP in organic catalysis, and Figure 14 presents chosen structures of silica-Fe_3_O_4_-based SILLPs.

**Figure 15:**
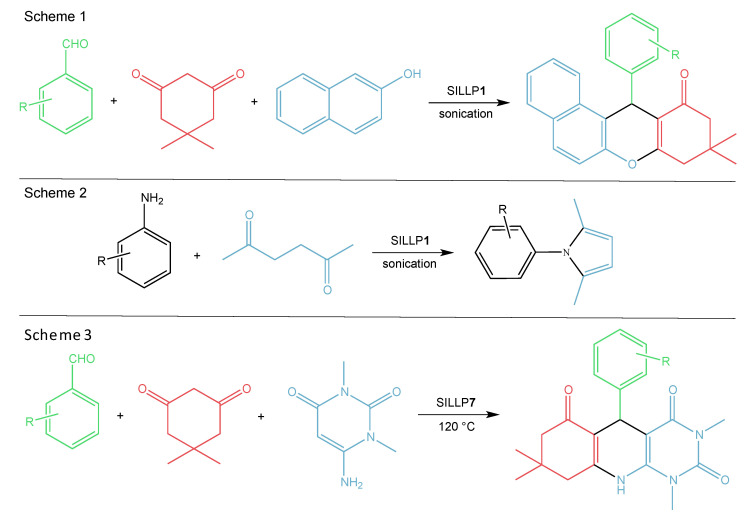
Silica-ferrite hybrid-based SILLP as the catalyst in synthesis of benzoxanthenes (Scheme 1), pyrroles (Scheme 2), pyrimido[4,5-b]quinolines (Scheme 3).

A Lewis chloroaluminate IL was grafted to a SiO_2_⋅Fe_3_O_4_ nanomaterial, and the catalytic activity of obtained SILLP **1** (Figure 14) was tested in the synthesis of β-keto enol ethers. The magnetic catalyst showed proper efficiency and provided high yields under mild reaction conditions. Moreover, the SILLP maintained activity for six reaction cycles, and its recovery through external magnetic field was very effective [94]. The next report examined Lewis magnetic SILLP **1** (Figure 14) based on the chlorozincate (II) anion in the synthesis of benzoxanthenes (Scheme 1, Figure 15) and pyrroles (Scheme 2, Figure 15). In both reactions, the SILLP presented excellent activity, reusability for 5 runs, and achieved 76–96% yields of benzoxanthenes and pyrroles. In comparison with the described catalysts, the magnetic SILLP is a promising alternative due to its versatility [95]. Brønsted hydrogensulfate IL was anchored to magnetic silica-based material, where SILLP **2** (Figure 14) exhibited excellent activity and achieved 87–97% yields in the condensation reaction of cyclic diketones with aromatic aldehydes and ammonium acetate or primary amines. The catalyst could be reused nine times, which additionally proves the wide applicability of this nanomaterial [96]. In another report, SILLP **2** (Figure 14) found application as a catalyst for the thiocyanation of aromatic and heteroaromatic compounds. High yields of 88–98%, regioselectivity, short reaction times, and reusability (seven runs) were achieved [97]. The next report described the magnetic SILLP **3** (Figure 14) based on the phenyltetrazole cation. The SILLP nanocatalyst examined its efficiency in the synthesis of antibacterially active 1-carbamoyl-1-phenylureas in water. The magnetic nanomaterial gave 89–93% yields of the main products and kept good catalytic activity during five reaction cycles [98]. Hydrogensulfate poly(ionic liquid) was grafted to silica magnetic nanoparticles via the polymerization of vinylimidazolium moieties. The catalytic activity of the prepared heterogeneous catalyst was checked in the acetylation of aldehydes with acetic anhydride, which resulted in 90–98% yields and 10 reaction cycles without activity loss. Moreover, the SILLP also showed good efficiency in the deprotection reaction of acyl [99]. The Brønsted triethylenediammonium ditriflate based magnetic SILLP **4** (Figure 14) was found to be a great catalyst in the synthesis of 3,3-di(indolyl)indolin-2-ones. A yield of 85–96% of various indolines compounds with medical properties and eight efficient catalytic cycles were achieved with SILLP **4** [100]. In other work, Fe_3_O_4_ nanoparticles coated with silica SILLP **2** (Figure 14) based on the dihydrogenphosphotungstate anion catalyzed the synthesis of tetrahydrodipyrazolo-pyridines. This catalytic system could be reused several times using magnetic external forces and high loadings of the IL, providing excellent yields (90–98%) under mild conditions [101]. Further reports present Dual Brønsted acidic ILs immobilized on silica coated magnetic nanoparticles. SILLP **5** (Figure 14) found applications as a catalyst in the synthesis of benzoxanthenes [102], spirooxindoles [103], and biodiesel production from oleic acid [104,105]. This novel catalyst demonstrated great versatility and activity in all mentioned processes, achieving high yields for benzoxanthenes (84–91%), spirooxindoles (81–90%), biodiesel (90–94%) synthesis, as well as short reaction times, high products quality, easy catalyst recovery via magnetic field, and great reusability, which makes SILLP **5** very attractive for industrial use [102,103,104,105]. In other work, a phosphonium-SO_3_H based IL was anchored to the magnetic silica nanomaterial, creating the Brønsted-type SILLP **6** (Figure 14) catalyst. Its activity was tested in the acetalization of aldehyde or ketone with ethylene glycol, which resulted in high product yields of 94–97% with various substrates, and the possibility of SILLP catalyst recycling five times without significant loss of activity [106]. Interestingly, the dicationic IL grafted to magnetic nanoparticles (**7**, Figure 14) found application in the synthesis of pyrimido[4,5-b]quinolines (Scheme 3, Figure 15). The novel SILLP **7** hydrogensulfate anion provides one acidic hydrogen and one weakly basic (negative oxygen) site, and was successfully used in the synthesis that requires an acidic and a basic catalyst. This magnetic catalytic system performed well, with yields of 81–96%, short reaction times, and recovery for four reaction cycles, with only a slight decrease in activity [107].

A follow-up report described *N*-(propyl-triethoxysilane)-2-pyrrolidinium hydrogensulfate immobilized on Fe_3_O_4_ silica nanoparticles (**8**, Figure 14) as an efficient catalyst for the one-pot diazotization–halogenation of the aromatic amines. Utilization of SILLP **8** as a green catalyst turned out to provide satisfying yields and short reaction times [108]. Silica coated cobalt ferrite nanoparticles were modified with a 3-(4-sulfobutyl)-1-(3-trimethoxysilylmerkaptopropyl)imidazolium triflate IL, and were used in the esterification of oleic acid with straight-chain alcohols. Higher SH-group loading on the silica surface resulted in a decreasing pore diameter and surface area. On the other hand, however, less IL moieties could be immobilized on the surface of the nanomaterial. Increasing the alkyl chain in the alcohol caused mass transfer resistance, which resulted in a decreased conversion. This kind of SILLP could find application in shape-selective catalysis [109]. Other work described a sulfo-tetrazolium hydrogensulfate based IL anchored to magnetic nanoparticles. Its activity was tested in the one-pot synthesis of pyrimidine derivatives under mild conditions. The catalyst provided 80–95% yields, an easy separation method using magnetic forces, and could be recycled for six reaction cycles without any activity loss. Moreover, in comparison with another catalyst described in literature, this SILLP is an outstanding green alternative [110]. 2-hydroxyethylammonium sulphonate IL was immobilized via the anion on a magnetic silica-based material. The catalyst possesses basic sites such as hydroxyl groups and acidic sites such as ammonium moieties, and was therefore successfully used in the one-pot three-component synthesis of 2-amino-3,5-dicarbonitrile-6-thio-pyridines. Satisfying yields of 81–91% of various pyridines were achieved, as well as a reusability of five reaction cycles in the reaction between benzaldehyde, malononitrile, and thiophenol characterized this catalyst as very efficient [111]. More recent work was also carried out on a 1,4-diazabicyclo[2.2.2]octane-based basic IL immobilized on silica coated ferrite nanomolecules for a Knoevenagel condensation. The SILLP showed excellent catalytic performance, high yields, short reaction times, and could be reused for eight times. Specific activity could be explained with synergistic action of the tertiary amine, IL, and nanoparticles [112]. Further reports concern the applications of Fe_3_O_4_-silica nanoparticles modified with 1-methyl-3-(triethoxysilylpropyl)imidazolium chloride. SILLP **9** (Figure 14) catalytic activity was investigated in the synthesis of 1,3-thiazolidin-4-ones [113], indole-substituted pyrido[2,3-d]pyrimidines [114], 3,4-dihydropyrimidin-2(1H)-ones/thiones [116], cycloaddition of CO_2_ to epoxides [115], and N-formylation of amines [117]. As shown in Table 3, high reaction yields, easy catalyst recovery, and the possibility of recycling make SILLP **9** not only versatile, but also very efficient. The same catalyst type, but with a triazinium cation, was tested for the synthesis of 4H-dihydropyrano[3,2-b]pyran-3-carbonitrile derivatives. High yields of 85–98% were achieved for various benzaldehydes (Table 3), and newly synthesized compounds indicate potential antioxidant and antifungal properties. Furthermore, this catalyst could be reused four times without any loss of activity [118]. Next, a 3-((3-(trisilyloxy)propyl)propionamide)-1-methylimidazolium chloride IL anchored to silica magnetic nanoparticles was used in the acetylation of alcohols with acetic anhydride under mild conditions. Good yields (93–96%), simple separation by magnetic decantation, and reusability for nine cycles without activity loss were reported for this SILLP [119]. The SILLP magnetic nanoparticles formed from imidazolium-aniline based IL were applied in the Mannich reaction between arylaldehydes, anilines, and cyclohexanone under ultrasound irradiation. The catalyst provided high yields of the main product, high diastereoselectivity (anti:syn), short reaction times, and could be easy reused six times without activity loss, which makes it competitive to previous achievements in this field [120]. In another report, a basic 1-triethylamineimidazolium based IL immobilized on silica coated magnetic nanoparticles was tested for Knoevenagel condensation between various aldehydes and malonitrile. As shown in Table 3, high yields and five reaction cycles with this magnetic SILLP were achieved. In comparison, the IL was immobilized on polystyrene-divinylbenzene resin, but the magnetic silica-based SILLP showed better activity than the polymeric one, presumably due to a more basic character of the silica-ferrite matrix [121]. Studies on immobilized triethyltryptophanium iodide IL on titanomagnetite silica matrix as the catalyst in the synthesis of 6-substituted quinolinedialkyl-2,4-dicarboxylates showed that the library of compounds achieved good yields, the possibility of convenient catalyst recovery, and reusability for three reaction runs were reached in the presence of the SILLP [122].

### 3.4. SILLP as Matrix for Metals, Organocatalysts, and Enzymes

The catalytic features of the developed SILLPs applications as a matrix or co-catalyst are known, and there are many reports of the use of an SILLP as a matrix/co-catalyst for metal particles, organocatalysts, or enzymes. In Table 4, only examples of silica-based SILLP applications as a matrix are shown due to existing accurate reviews on this topic [34,39,40,41,123].

The hydroformylation reaction is one of the firsts reports on the application of silica based-SILLP as a matrix for metal-based catalysts. Rh particles were introduced to the SILLP with a ligand (to prevent any leaching of Rh) and used for *n,i*-heptanal production with TOF = 65 min^−1^, which, compared to the typical biphasic IL approach (TOF = 23 min^−1^), was a major accomplishment. The catalyst owes its higher activity to a higher concentration of Rh particles on the surface, as well as a larger surface area. Further studies on this topic included physical adsorption of ILs on solid supports (SILP) as Rh particles matrix and continuous-flow processes, which is more accurately described by Haumann in the review [37,124,125,126]. Next, research shows a novel bifunctional Ni-IL/SiO_2_ in 2-propylheptanol synthesis through a one-pot, self-condensation and hydrogenation from *n*-valeraldehyde. Ils possess a Brønsted -SO_3_H group and act like both a matrix and a co-catalyst. With the Ni-SILLP catalytic system, 100% conversion, 75.4% selectivity of the main product, and 98.6% production of 2-propylheptanol and pentanol were achieved [127]. Other reports present SILLP as effective matrices for PbS nanoparticles. A high surface area and IL presence enabled high loading of PdS molecules without aggregation. Additionally, synergistic effects between metal-based particles and Ils provided great catalytic activity in the dehydrogenation of formic acid, with 100% degradation of the acid, 78% selectivity to hydrogen, and TOF = 604 h^−1^ [128]. Further studies present immobilization and stabilization of Pd particles via SBA-15-based SILLP. New versatile and efficient catalytic systems were tested in the Suzuki coupling and Heck reactions. As shown in Table 4, the library of compounds was synthesized with high yields, and a catalyst could be recycled several times. Applications of SBA-15 with hexagonal pores as a matrix, which behaved as nanoreactors, assured excellent catalytic activity [129,130,131]. Moreover, transition from a batch to a continuous process provided conversion of 27 g of substrate to the main product using only 42 mg of the Pd-SILLP catalyst as well as reducing waste, which significantly reduced the E-factor [131]. The next report shows a silica-based SILLP as a carrier for the *cis*-ion-tagged proline. Proline moieties dissolved in covalently immobilized multilayered IL film performed with excellent activity in an asymmetric aldol reaction. The catalyst provided high yields and enantioselectivity of the main products (Table 4) and could also be recycled up to 15 times [132]. SILLPs can also be used for enzyme immobilization. Moreover, many reports confirmed an IL stabilizing effect on three-dimensional structures of enzymes, increasing protein activity. For example, the catalytic activity of lipase from *Candida antarctica* (CALB) adsorbed on an imidazolium silica-based SILLP was examined in corn oil glycerolysis to diacylglycerol production. The presence of the IL resulted in increasing the catalytic activity from 1855 to 5044 U/g and selectivity from 3.72 to 11.99 (ratio of diacylglycerols/monoacylglycerols). Additionally, the biocatalyst could be recycled for five reaction cycles, and even retained its activity at 50 °C [133]. Another lipase from *Porcine pancreas* immobilized on the same SILLP matrix was used in triacetin hydrolysis. Immobilized enzyme exhibited extremely high thermal stability, where even at 65 °C activity loss did not occur [134]. Lipase from *Candida rugosa* (CRL) was adsorbed on magnetic silica nanoparticles and used in the production of trans-free plastic fats. CRL-SILLP bionanomaterial catalyzed interesterifications of solid palm stearin and liquid rice bran oil for product possesses desirable physicochemical properties. In this case, convenient separation of the biocatalyst enabled its recycling up to four times [135].

## 4. Conclusions

In summary, achievements in the use of silica-based supported ionic liquid-like phases in heterogeneous organic catalysis were presented. Many Lewis and Brønsted acidic ionic liquids were found to be extremely active as heterogeneous catalysts. For the synthesis of Lewis type silica-based SILLP, chloroaluminate (III), chlorogallate (III), chloroferrate (III), chloroindate (III), chlorostannate (II), chlorozincate (II) anions or hydroxysulfonyl/chlorosulfonyl groups in the cation alkyl chain were used. In case of the forming of Brønsted-type silica-based SILLP, hydrogensulfate, dihydrogenphosphate, dihydrogenphosphotungstate anions, and/or sulfoalkyl group on the cation were found. Replacement of the halogen anions should be further investigated to prevent hydrolysis and the formation of hazardous acids as HCl. This would result in the reduction of costs, toxic waste, specific equipment, and apparatus corrosion. The anion of the ionic liquid has a crucial influence on the SILLP properties—the synthesized catalyst could be more or less acidic depending on the specific requirements, therefore application of SILLP as a catalyst is very convenient. For the SILLP synthesis, various cations such as imidazolium, alkylammonium, phosphonium, pyrrolidinium, tetrazolium, diammonium, triazinium, and tryptophanium were used, though their selection depended mainly on the substrate or nature of the reaction. It was mainly acidic ILs that were anchored to the silica surface that exhibited great catalytic activity and reusability, and from this the heterogeneous catalyst recovery was very easy. By cross-referencing the presence of the homogeneous and heterogeneous catalysis in the ionic liquids, it can be concluded that IL immobilization increases its catalytic activity due to enhanced mass transfer and availability of active sites. The selection of the silica material also brings many options in terms of size, shape, and density of pores, and hydroxyl groups on the surface. Silica materials such as SBA-15 and MCM-41, with their well-ordered, regular, and hexagonal array of pores, form microreactors that enhance the process efficiency. Obviously, the most important feature is the simplicity of the chemical modification of the surface via trimethoxysilyl/triethoxysilyl groups present as IL precursors, e.g., (3-(chloropropyl)triethoxysilane, 3-(chloropropyl)trimethoxysilane, 3-mercaptopropyltrimethoxysilane, or (3-aminopropyl)-trimethoxysilane). Moreover, the silica can be doped with ferrate nanoparticles, giving the surface magnetic properties. These magnetic-silica nano-catalysts can be removed and recycled by applying external magnetic forces, which is a very convenient approach. Additionally, the magnetic separation also increases the product purity and quality. It should be noted, however, that few examples of the continuous processes with silica-based SILLP have been developed. Flow catalysis offers many advantages compared to batch processes, for example: waste reduction, optimization of pure product synthesis and isolation, reduction of the amount of solvent required, and optimization of the catalyst recovery and recycling. Continuous catalysis simply means an efficient process, as well as green and environmentally friendly production, which is very attractive to the chemical industry. Silica-based SILLPs are versatile, stable catalysts, easy to synthesize, and reusable, with big potential for continuous-flow processes. SILLPs are also potential candidates for the development of sustainable and green chemical processes.

## Figures and Tables

**Table 1:** Lewis type silica-based SILLP in organic catalysis.

Catalyst	Reaction Type	Reaction Conditions	Reaction Parameters	Lit.
SiO_2_ [tespmim][Cl-AlCl_3_] ^a^	Friedel–Crafts alkylation of benzene with dodecene	6% wt. cat., benzene:dodecene (10:1; n/n), 80 °C, 1 h	A ^b^ > 90%, S ^c^ > 90%	[29]
SiO_2_ [pmim][Cl-AlCl_3_] ^d^	Friedel–Crafts alkylation of benzene with olefins	1% wt. cat., benzene:olefin (10:1; n/n), 20 °C, 2 h	C_6_: α = 45.3%, S = 73.8% C_8_: α = 44.9%, S = 96.5% C_10_: α = 34.1%, S = 89.6% C_12_: α = 35.2%, S = 80.3% (for 6% wt. cat., 80 °C, 1 h; α = 99.4%, S = 99.7%)	[50]
SiO_2_ [tms(p)_4_N][Cl-SnCl_4_] ^e^	Condensation of isobutene and formaldehyde	4% mol of SnCl_4_, isobutene:formaldehyde (1:0.1; n/n), chloroform 26 mL, 60 °C, 2 h	α = 76%, S = 94%, Y ^f^ = 71.4%, TON ^g^ = 2.63⋅10^−3^ s^−1^	[51]
SiO_2_ [p(p-SO_2_Cl)im][OTf] ^h^	Synthesis of bis(indolyl)methanes	143 mg cat., aldehyde 0.3 mmol, indole 0.5 mmol, MeCN 3 mL, rt, 1.5–9 h	Yields for: bezaldehyde 97%, p-nitrobenzaldehyde 97%, p-chlorobenzaldehyde 90%, p-acetoxybenzaldehyde 64%, p-methoxybenzaldehyde 97%, hydrocinnamaldehyde 98%	[52]
SiO_2_ [p(p-SO_2_Cl)im][OTf]	Esterification of acetic or decanoic acid with alcohols	Mole ratio of carboxylic acid to ionic liquid: 350, alcohol 20 mmol, carboxylic acid, 10 mmol, 100 °C, 8 h	Yields for various alcohols: (a) acetic acid: C_8_H_17_ 94.6%, C_10_H_21_ 95.1% (b) decanoic acid: C_2_H_5_ 86.3%, C_10_H_21_ 90.4%	[53]
SiO_2_ [p(p-SO_2_OH)im][OTf] ^i^	Nitration of aromatic compounds	Mole ratio of aromatic compound:ionic liquid: 20, mole ratio of aromatic compound:nitric acid: 1:3, 80 °C, 4 h	Conversions for R-groups in aromatic ring: H 61.6%, Me 85.8%, Cl 10.4%, Br 22.2%	[53]
SiO_2_ [p(p-SO_2_Cl)im][OTf]	Addition of indole to vinyl ketones	171 mg cat., vinyl ketone 0.6 mmol, indole 0.3 mmol, Et_2_O 0.2 mL, rt, 1.5–9 h	Yields for various ketones: 1-penten-3-one 92%, 2 2-cyclopentenone 88%, 3-penten-2-one 90%, benzalacetone 72%, dibenzylideneacetone 93%	[54]
SiO_2_ [tespmim][Cl-AlCl_3_]	Production of alkylated gasoline	0.5 g cat., iC4/C4 = 20, 80 °C, 90 min	α = 97%, S_C8_ = 59.7%	[55]
SiO_2_ [tespmim][Cl-AlCl_3_]	Trimerization of isobutene	30% wt. cat., isobutane:isobutene molar ratio 10:1, 25 °C, 600 h^−1^	α = 91.4%, S_C12_ = 79.4%	[56]
SiO_2_ [tespmim][Cl-FeCl_3_] ^j^	Friedel–Crafts reaction between benzene and benzyl chloride	0.05 g cat., benzene:benzyl chloride molar ratio 10:1, benzyl chloride 0.32 g, 80 °C, 45 min	α = 100%, S = 100%, 10 cycles	[57]
SiO_2_ [(tesp)_2_im][Cl-InCl_3_] ^k^	Friedel–Crafts reaction between benzene and benzyl chloride	0.05 g cat., benzene:benzyl chloride molar ratio 10:1, benzyl chloride 0.32 g, 80 °C, 15 min	α = 100%, S = 100%, 6 cycles	[58]
SiO_2_ [tespmim][Cl-GaCl_3_] ^l^	Diels–Alder cycloaddition of cyclopentadiene to various dienophiles	5% mol of GaCl_3_, cyclopentadiene:dienophile (12:8; n/n), 25 °C, 5–30 min	Methyl acrylate: α = 99%, endo:exo ratio: 95:5, 4 cycle; ethyl acrylate: α = 99%, endo:exo ratio: 93:7; diethyl maleate: α = 99%, endo:exo ratio: 93:7; methacrolein: α = 100%, endo:exo ratio: 80:20; benzoquinone: α = 83%; maleic anhydride: α = 89%	[59]
SiO_2_ [tespmim][Cl] ^m^	Cycloaddition of CO_2_ to styrene oxide	0.5% mol cat., 0.1% mol ZnBr_2_, styrene oxide 0.13 mol, 100 °C, P_CO2_ = 1 MPa, 6 h, 700 rpm	α = 83%, Y = 72%	[60]
SiO_2_-Zn [tespmim][Cl]	Cycloaddition of CO_2_ to propylene oxide	S/C = 200 (PO mol per cat. mol), V_PO_ = 8 mL, P_CO2_ = 1.25 MPa, 100 °C, 8 h	MCM-41: α = 33%, S = 98% MSN: α = 76%, S = 97% BMMs: α = 77%, S = 98%	[61]

^a^ 1-methyl-3-(triethoxysilylpropyl)imidazolium chloride—chloroaluminate (III), ^b^ conversion, ^c^ selectivity, ^d^ 1-propyl-3-methylimidazolium chloroaluminate (III) immobilized via anion, ^e^ 3-trimethoxypropyltripropylammonium chloride—chlorostannate (IV), ^f^ yield, ^g^ turnover number, ^h^ 1-(3-chlorosulfonylpropyl)-3-(3-trimethoxysilylmerkaptopropyl)imidazolium trifluoromethanesulfonate (triflate), ^i^ 1-(3-hydroxysulfonylpropyl)-3-(3-trimethoxysilylmerkaptopropyl)imidazolium triflate, ^j^ 1-methyl-3-(triethoxysilylpropyl)imidazolium chloride—chloroferrate (III), ^k^ 1-(triethoxysilylpropyl)-3-(triethoxysilylpropyl)imidazolium chloride—chloroindate (III), ^l^ 1-methyl-3-(triethoxysilylpropyl)imidazolium chloride—chlorogallate (III), ^m^ 1-methyl-3-(triethoxysilylpropyl)imidazolium chloride.

**Table 2:** Brønsted-type silica-based SILLP in organic catalysis.

Catalyst	Reaction Type	Reaction Conditions	Reaction Parameters	Lit.
SiO_2_ [p(b-SO_3_H)im][OTf] ^a^	Estrification of oleic acid and methanol	0.2 mmol IL in cat., oleic acid 17.7 mmol, methanol 531.05 mmol, 100 °C, 4 h	α = 84%, 3 cycles	[62]
SiO_2_ [p(b-SO_3_H)im][OTf]	Transestrification of glicerol trioleate and methanol	0.2 mmol IL in cat., glicerol trioleate 17.7 mmol, methanol 531.05 mmol, 100 °C, 4 h	α = 30%, S_methyl oleate_ = 36%	[62]
SiO_2_ [tesp(b-SO_3_H)im][Cl] ^b^	Hydrolysis of cellulose	0.02 mmol H^+^ in cat., cellulose 0.185 mmol, 2 mL H_2_O, 190 °C 3 h	Y_TRS_ = 48.1%, Y_glucose_ = 21.9%, 4 cycles	[63]
SiO_2_ [p(p-SO_3_H)im][OTf] ^c^	Dehydration of fructose	0.175 mmol IL in cat., fructose 0.35 mmol, DMSO 2.0 g, MW: 200 W, 4 min	α = 100%, Y = 70.1% (5-hydroxymethylfurfural)	[64]
SiO_2_ [tmsp(p-SO_3_H)im][I] ^d^	Biginelli reaction	0.8% mol cat., aldehyde 1 mmol, ethyl/methyl-acetoacetate 1 mmol, urea 1.5 mmol, 75 °C, 50–90 min	Yields for aldehydes with Ar groups: (a) ethylacetoacetate: Ph 96%, 4-OMeC_6_H_4_ 97%, 2-OMeC_6_H_4_ 86%, 4-MeC_6_H_4_ 96%, 4-ClC_6_H_4_ 90%, 3-BrC_6_H_4_ 96% (b) methylacetoacetate: Ph 96%, 4-OMeC_6_H_4_ 96%, 2-OMeC_6_H_4_ 89%, 4-MeC_6_H_4_ 95%, 4-ClC_6_H_4_ 92%, 3-BrC_6_H_4_ 95%	[65]
SiO_2_ [tmsp(p-SO_3_H)im][I]	Esterification of acetic acid with various alcohols	5% mol cat., alcohol 2 mmol, acetic acid 4 mmol, 60–70 °C, 12–24 h	Yields for alcohols: PhCH_2_OH 95%, PhCH(OH)CH_3_ 86%, PhCH(OH)CH_2_CH_3_ 85%, PhCH_2_CH_2_OH 88%, CH_3_CH_2_OH 93%, C_8_H_17_OH 93%, C_9_H_19_OH 92%, C_10_H_21_OH 92%	[66]
SiO_2_ [tesp(p-SO_3_H)im][OTf] ^e^	Self-condensation of pentanal	10% wt. cat, 120 °C, 6 h	α = 77.4%, Y = 69.4%, S = 89.6%, TON = 230.5	[67]
SiO_2_ [tesp(p-SO_3_H)bim][Cl] ^f^	Synthesis of fatty acid methyl esters	3% wt. cat., castor/jatropha/neem oil:methanol 1:12 molar ratio, 70 °C, 6–7 h	Castor oil Y = 94.9% Jatropha oil Y = 95.7% Neem oil Y = 94.4%	[68]
SiO_2_ [tesp(p-SO_3_H)bim][Cl]	Synthesis of 1-amidoalkyl naphthols	80 mg cat., aldehyde 20 mmol, 2-naphthol 20 mmol, acetamide 24 mmol, 100 °C, 7–10 h	Yields for benzaldehydes with R group: H 90%, 3-NO_2_ 95%, 4-OH 87%, 4-OMe 89%, 2-Cl 92%, 4-Cl 93%, 4-NO_2_ 89%	[69]
SiO_2_ [tespmim][HSO_4_] ^g^	Bayer–Villiger oxidation of cyclic ketones	0.4 g cat., ketone 1 mmol, 68% H_2_O_2(aq_._)_ 3 mmol, dichloromethane 4 mL, 50 °C, 5–15 h	cyclobutanone: α = 100%, Y = 96%; cyclopentanone: α = 98%, Y = 75%; cyclohexanone: α = 86%, Y = 64%; 2-adamantanone: α = 95%, Y = 89%; 1-indanone: α = 81%, Y = 78%; 1-tetralone: α = 78%, Y = 77%	[70]
SiO_2_ [tespmim][HSO_4_]	Esterification of acetic acid and butanol	4% wt. cat., 96℃, butanol 0.12 mol, acetic acid 0.10 mol, cyclohexane 6 mL, 3 h	α = 99.4%, 6 cycles	[71]
SiO_2_ [tespmim][HSO_4_]	Synthesis of 1-(benzothiazolylamino) phenylmethyl-2-naphthols	150 mg cat., aldehyde 1 mmol, 2-aminobenzothiazole 1 mmol, 2-naphthol 1 mmol, 110 °C, 3–5 h	Yields for various aryl aldehydes with R-groups: H 93%, 2-Cl 89%, 4-Cl 92%, 3-Br 93%, 4-Br 91%, 3-Me 93%, 2-OMe 90%, 3-OMe 92%, 4-OMe 93%, 2-NO_2_ 90%, 3-NO_2_, 92%, 4-NO_2_ 91%	[72]
SiO_2_ [tespmim][HSO_4_]	Formylation of amines	0.8% mol cat., amine 1 mmol, formic acid 2 mmol, 60 °C, 1–15 h	Yields and TOF for amines: aniline 94%, 1428 h^−1^; 4-methoxy aniline 97%, 7275 h^−1^; benzyl amine 93%, 465 h^−1^	[73]
SiO_2_ [tespmim][HSO_4_]	Knoevenagel–Michael cyclization for polyhydroquinolines synthesis	2% mol cat., aldehyde 1 mmol, dimedone 1 mmol, enaminone 1.2 mmol, NH_4_OAc 1.5 mmol, H_2_O 3 mL, 45 °C, 2–3 h	Yields for enaminone-COOMe with aldehydes with Ar-group: C_6_H_5_ 90%, 4-C_6_H_5_ 93%, 2-C_6_H_5_ 92%, 4-OMeC_6_H_5_ 88%, 2-MeC_6_H_5_ 90%	[74]
SiO_2_ [tespmim][HSO_4_]	Synthesis of 3,4-dihydropyrano[c] chromenes	0.15 g cat., 4-hydroxycoumarin 1 mmol, malononitrile 1 mmol, Ar-aldehyde 1 mmol, 100˚C, 30 min	Yields for aldehydes with Ar-groups: C_6_H_5_ 94%, 4-ClC_6_H_4_ 95%, 3-ClC_6_H_4_ 93%, 4-BrC_6_H_4_ 94%, 2,4-(Cl)_2_C_6_H_3_ 90%, 3-O_2_NC_6_H_4_ 93%, 4-O_2_NC_6_H_4_ 90%, 2-O_2_NC_6_H_4_ 89%, 4-MeC_6_H_4_ 94%, 3,4,5-(CH_3_O)_3_C_6_H_2_ 89%, 4-HO-C_6_H_4_ 93%	[75]
SiO_2_ [tespmim][HSO_4_]	Synthesis of pyrano[3,2-b]indole derivatives	10% mol cat., 3-hydroxypyrrole 1 mol, benzaldehyde, 1 mol, malononitrile 1 mol, acetonitrille 8 mL, 80 °C, 6–8 h	Yields for aldehydes with Ar groups: 4-CH_3_OC_6_H_4_ 84%, C_6_H_5_ 90%, 4-CH_3_C_6_H_4_ 85%, 4-BrC_6_H_4_ 90%, 2-BrC_6_H_4_ 86%, 4-ClC_6_H_4_ 90%, 2-ClC_6_H_4_ 86%, 4-CNC_6_H_4_ 90%, 4-NO_2_C_6_H_4_ 85%, 2-NO_2_C_6_H_4_ 88%	[76]
SiO_2_ [tespmim][HSO_4_]	Synthesis of pyrano[2,3-b]pyrrole derivatives	10% mol cat., 2-hydroxypyrrole 1 mol, benzaldehyde 1 mol, malonoitrile 1 mol, acetonitrille 4 mL, 60 °C, 2–8 h	Yields for aldehydes with Ar groups: 4-CH_3_OC_6_H_4_ 76%, C_6_H_5_ 90%, 4-CH_3_C_6_H_4_ 82%, 4-BrC_6_H_4_ 90%, 2-BrC_6_H_4_ 88%, 4-ClC_6_H_4_ 90%, 2-ClC_6_H_4_ 86%, 4-CNC_6_H_4_ 73%, 2-CNC_6_H_4_ 70%, 4-NO_2_C_6_H_4_ 64%, 2-NO_2_C_6_H_4_ 62%	[77]
SiO_2_ [tespmim][HSO_4_]	Synthesis of benzo[f]chromene compounds	15% mol cat., 2-naphthol 1 mol, benzaldehyde 1 mol, triethyl orthobenzoate 1 mol, acetonitrille 4 mL, 65 °C, 4–8 h	Yields for benzaldehydes with 4-group: H 85%,Br 85%, Cl 88%, NO_2_ 80%, Me 88%, OMe 90%, OH 84%	[78]
SiO_2_ [tespmim][HSO_4_]	Synthesis of 2,9-dihydro-9-methyl-2-oxo-4-aryl-1H-pyrido[2, 3-b]indole-3-carbonitrile compounds	15% mol cat., 1-methyl-1H-indol-2-ol 1 mol, (triethoxymethyl)arene 1 mol, cyanoacetamide 1 mol, DMF 6 mL, 100 °C, 2–7 h	Yields for (triethoxymethyl)arene with groups: 4-OMe 73%, H 65%, 4-Me 65%, 4-Br 61%, 2-Br 56%, 4-Cl 61%, 2-Cl 55%, 4-F 53%	[79]
SiO_2_ [tespmim][HSO_4_]	Synthesis of acenaphtho[1,2b]pyrroles.	10% mol cat., silyl enol of acenaphthylen-1(2H)-one 1 mol, 2,4-dimethoxybenzaldehyde 1 mol, isocyanocyclohexane 1 mol, DMF 50 mL, reflux, 10 h	Y = 97%	[80]
SiO_2_ [tespmim][HSO_4_]	Synthesis of 5-Amino-7-aryl-6-cyano-4H-pyrano[3,2-b]pyrroles	10% mol cat., 3-hydroxypyrrole 1 mol, aldehyde 1 mol, malononitrile 1 mol, acetonitrille 4 mL, 50 °C, 1–8 h	Yields for aldehydes with Ar groups: 4-CH_3_OC_6_H_4_ 62%, C_6_H_5_ 89%, 4-CH_3_C_6_H_4_ 80%, 4-BrC_6_H_4_ 91%, 2-BrC_6_H_4_ 89%, 4-ClC_6_H_4_ 88%, 2-ClC_6_H_4_ 88%, 4-CNC_6_H_4_ 70%, 2-CNC_6_H_4_ 67%, 4-NO_2_C_6_H_4_ 61%, 2-NO_2_C_6_H_4_ 69%	[81]
SiO_2_ [tespmim][H_2_PW_12_O_40_] ^h^	Oxidation of dibenzothiophene	0.01 g cat., O/S molar ratio: 3:1 (H_2_O_2_ 0.8 mmol), 60 °C, 40 min	α = 100%, 4 cycles	[82]
SiO_2_ [p(p-SO_3_H)im][HSO_4_] ^i^	Esterification of acetate acid and n-butanol	6% wt. cat., n-butanol:acetic acid (2:1, n/n), 94 °C, 3 h	Y = 99.5%	[83]
SiO_2_ [tesp(b-SO_3_H)im][HSO_4_] ^j^	Synthesis of amidoalkyl naphtols	80 mg cat., aldehyde:2-naphtol:acetamide (2:2:2.4; n/n/n), 85 °C, 5–15 min	Yields and TOF for different aldehydes with R-groups: Ph 90%, 6.43 min^−1^; 4-Cl–C_6_H_4_ 89%, 3.18 min^−1^; 2,4-Cl_2_–C_6_H_3_ 86%, 3.84 min^−1^; 4-Br–C_6_H_4_ 88%, 3.15 min^−1^; 3-NO_2_–C_6_H_4_ 92%, 6.59 min^−1^; 4-NO_2_–C_6_H_4_ 93%, 6.65 min^−1^; 3-MeO–C_6_H_4_ 86%, 3.07 min^−1^; 4-MeO–C_6_H_4_ 80%, 1.91 min^−1^; 4-Me–C_6_H_4_ 87%, 3.11 min^−1^	[84]
SiO_2_ [tesp(b-SO_3_H)im][HSO_4_]	Thioacetalization of carbonyl compounds	5% mol cat., 4– methoxybenzaldehyde with thiophenol, rt, 5 h	Y = 96%, 6 cycles	[85]
SiO_2_ [tesp(b-SO_3_H)im][HSO_4_]	Acetalization of benzaldehyde or furfural with diols	4% wt. cat., benzaldehyde 70 mmol, ethanediol 126 mmol, cyclohexane 8 mL, reflux, 1.5–3 h	Yields: (a) benzaldehyde: ethanediol 95.2%, 1,2-propanediol 93%, 1,4-Butanediol 87.1% (b) furfural: ethanediol 85%, 1,2-propanediol 95.9%	[86]
SiO_2_ [tesp(p-SO_3_H)im][HSO_4_]	Synthesis of 2H-indazolo[1,2-b]phthalazine-triones	30 mg cat., benzaldehyde 1 mmol, dimedone 1 mmol, phthalhydrazide 1 mmol, 80 °C, 10 min	Y = 94%, 8 cycles	[87]
SiO_2_ [tesp(b-SO_3_H)im][HSO_4_]	Synthesis of polyoxymethylene dimethyl ethers	4% wt. cat., molar ratio of methylal to trioxane 3, 105 °C, 1 h	α = 92%, S = 52%, 6 cycles	[88]
SiO_2_ [tesp(p-SO_3_H)im][HSO_4_] ^k^	Lignin depolymerization	0.5 g cat., dealkaline lignin 2% wt., 30 mL H_2_O:C_2_H_5_OH (1:5, *v*/*v*), 200 °C, 1 h	Yields for THF soluble products 90%,	[89]
SiO_2_ [p(p-SO_3_H)im][HSO_4_]	Esterification of acetic acid and n-butanol	8% wt. cat., acetic acid 4.8 g, n-butanol 7.12 g, cyclohexane 8 mL, 89 °C, 3 h	Y = 99.2%, S = 100%, 7 cycles; yields for other alcohols: C_6_H_13_ 99.4%, C_2_H_5_ 84.1%, C_6_H_5_CH_2_ 98.5%	[90]
SiO_2_ [tesp(p-SO_3_H)a][HSO_4_] ^l^	Biodiesel synthesis	0.05 g cat., rapeseed oil 5 g, methanol 2.33 g, 70 °C, 9 h	Y = 99%, 6 cycles	[91]
SiO_2_ [tesp(p-SO_3_H)a][HSO_4_]	Acetalization of benzaldehyde and 1,2-ethanediol	0.05 g cat., benzaldehyde 0.1 mol, 1,2-ethanediol 0.15 mol, 25 °C, 12 h	Y = 98%	[91]
SiO_2_ [tespim][H_2_PW_12_O_40_] ^m^	Oxidations of alkenes	0.05 g cat., alkene 5 mmol, hydrogen peroxide (30%) 5 mmol, acetonitrile 4.5 mL, 60 °C, 4 h	Conversion, selectivity and TOF for alkenes: cyclooctene 90%, 99%, 162 h^−1^; 1-octene 34%, 99%, 61 h^−1^; norbornene 85%, 99%, 153 h^−1^; limonene 76%, 29%, 137 h^−1^	[92]
SiO_2_ [p(p-SO_3_H)im] [H_2_PW_12_O_40_] ^n^	Esterification of palmitic acid	15% wt. cat., methanol:palmitic acid molar ratio 9, 65 °C, 8 h	Y = 88.1%, 5 cycles	[93]

^a^ 3-(4-sulfobutyl)-1-(3-trimethoxysilylmerkaptopropyl)imidazolium triflate, ^b^ 3-(4-sulfobutyl)-1-(3-propyltriethoxysilane)imidazolium chloride, ^c^ 3-(3-sulfopropyl)-1-(3-propyltriethoxysilane)imidazolium triflate, ^d^ 3-(3-sulfopropyl)-1-(3-propyltrimethoxysilane)imidazolium iodide, ^e^ 3-(3-sulfopropyl)-1-(3-propyltrimethoxysilane)imidazolium triflate, ^f^ 3-(3-sulfopropyl)-1-(3-propyltriethoxysilane)benzimidazolium chloride, ^g^ 1-methyl-3-(3-propyltriethoxysilane)imidazolium hydrogensulfate, ^h^ 1-methyl-3-(3-propyltriethoxysilane)imidazolium dihydrogenphosphotungstate, ^i^ 3-(3-sulfopropyl)-1-(3-trimethoxysilylmerkaptopropyl)imidazolium hydrogensulfate, ^j^ 3-(4-sulfobutyl)-1-(3-propyltriethoxysilane)imidazolium hydrogensulfate, ^k^ 3-(3-sulfopropyl)-1-(3-propyltriethoxysilane)imidazolium hydrogensulfate, ^l^ N-(3-sulfopropyl)-N-(3-propyltriethoxysilane)ammonium hydrogensulfate, ^m^ 1-(3-propyltriethoxysilane)imidazolium dihydrogenphosphotungstate, ^n^ 3-(3-sulfopropyl)-1-(3-trimethoxysilylmerkaptopropyl)imidazolium dihydrogenphosphotungstate.

**Table 3:** Silica-Fe_3_O_4_-based SILLPs in organic catalysis.

Catalyst	Reaction Type	Reaction Conditions	Technological Parameters	Lit.
SiO_2_⋅Fe_3_O_4_ [tmspmim][Cl-AlCl_3_] ^a^	Synthesis of β-keto enol ethers	0.27 g cat., 5,5-dimethylcyclohexane-1,3-dione 1 mmol, alcohol 3 mL, rt, 50–95 min	Yields for alcohols: methanol 94%, ethanol 93%, n-butanol 89%, n-pentanol 87%, 2-propanol 88%, cyclohexanol 86%	[94]
SiO_2_⋅Fe_3_O_4_ [tmspmim][Cl-ZnCl_2_] ^b^	Synthesis of benzoxanthenes	15 mg cat., benzaldehyde 1 mmol, 2-naphthol 1 mmol, dimedone 1 mmol, sonication, 80 °C, 30 min	Yields for benzaldehydes with R-groups: H 96%, 4-Me 84%, 2-OH 81%, 4-F 81%, 4-Cl 72%, 4-Br 76%, 2-F 70%, 2-Cl 75%, 2-Br 79%, 2-NO_2_ 90%	[95]
SiO_2_⋅Fe_3_O_4_ [tmspmim][Cl-ZnCl_2_]	Synthesis of pyrroles	15 mg cat., aniline 1 mmol, acetonylacetone 1.2 mmol, sonication, 30–90 min	Yields for anilines with R-groups: H 91%, 4-I 98%, 4-OH 95%, 2-OH, 5-Me 78%, 3,5-Cl 77%	[95]
SiO_2_⋅Fe_3_O_4_ [tmspmim][HSO_4_] ^c^	Synthesis of 1,8-dioxodecahydro-acridines	cyclic diketones: amines:aldehydes: catalyst (2:1:1:0.01), 80 °C, 10–30 min	Yields 87–97%	[96]
SiO_2_⋅Fe_3_O_4_ [tmspim][HSO_4_] ^d^	Synthesis of 3-thiocyanato-1H-indole	5 mg cat., indole:H_2_O_2_:KSCN (1:3:3; n/n), water:ethanol (1:4; *v*/*v*), rt	Y = 95%; for various substrates 88–98%	[97]
SiO_2_⋅Fe_3_O_4_ [tmsptetrazole-SO_3_H][Cl] ^e^	Synthesis of	0.2 g cat., arylcyanamide 1 mmol, NaOCN 1 mmol, H_2_O 10 mL, reflux	Yields for arylcyanamide with R-groups: 3-Br 90%, 4-Cl 89%, 4-Me 92%, 4-OMe 93%	[98]
SiO_2_⋅Fe_3_O_4_ [tmspim][HSO_4_]	1-carbamoyl-1-phenylureas	50 mg cat., benzaldehyde 1 mmol, acetic anhydride 5 mmol, rt, 10–120 min	Yields for benzaldehydes with R-groups: H 91%, 4-Cl 95%, 4-Me 93%, 4-OH 91%, 2-OH 97%, 4-MeO 90%, 2-MeO 87%, 4-COOH 90%, 4-CN 88%, 4-NO_2_ 98%	[99]
SiO_2_⋅Fe_3_O_4_ [tmspdabco(SO_3_H)] [OTf]_2_ ^f^	Acetylation of aldehydes with acetic anhydride	50 mg cat., isatin 0.5 mmol, indole 1 mmol, H_2_O 2 mL, 90 °C, 2 h	Y = 85–96%, 8 cycles	[100]
SiO_2_⋅Fe_3_O_4_ [tespmim][H_2_PW_12_O_40_] ^g^	Synthesis of 3,3-di(indolyl)indolin-2-ones	0.1 mg cat., hydrazine hydrate 2 mmol, ethyl acetoacetate 2 mmol, aryl aldehydes 1 mmol, ammonium acetate 3 mmol, water 15 mL, rt, 30 min.	Yields for different Ar-aldehydes: H 96%, Cl 95%, F 97%, NO_2_ 98%, OMe 92%, Me 93%, OH 90%, CN 95%	[101]
SiO_2_⋅Fe_3_O_4_ [tesp(b-SO_3_H)im][HSO_4_] ^h^	Synthesis of tetrahydrodipyrazolo-pyridines	55 mg cat., aldehyde 2 mmol, 2-naphthol 2 mmol, dimedone 2.4 mmol, 90 °C, 35–65 min	Yields for aldehydes with Ar groups: C_5_H_6_ 89%, 4-MeC_6_H_4_ 86%, 4-OMeC_6_H_4_ 84%, 4-ClC_6_H_4_ 91%, 3-ClC_6_H_4_ 84%, 4-BrC_6_H_4_ 90%, 3-BrC_6_H_4_ 88%, 4-NO_2_C_6_H_4_ 93%, 3-NO_2_C_6_H_4_ 90%, 2-NO_2_C_6_H_4_ 85%	[102]
SiO_2_⋅Fe_3_O_4_ [tesp(b-SO_3_H)im][HSO_4_]	Synthesis of benzoxanthenes	50 mg cat., isatin 1 mmol, 1,3-dimethyl-2-amino uracil 1 mmol, barbituric acid 1 mmol, H_2_O, 1 mL, rt, 4–8 h	Y = 81–90%, 5 cycles	[103]
SiO_2_⋅Fe_3_O_4_ [tmsp(p-SO_3_H)im][HSO_4_] ^i^	Synthesis of spirooxindoles	0.2 g cat., oleic acid 10 mmol, alcohol 60 mmol, 373K, 4 h	Methanol: Y = 89.6% Ethanol: Y = 93.5% n-propanol: Y = 92% n-butanol: Y = 91.5%	[104]
SiO_2_⋅Fe_3_O_4_ [tesp(p-SO_3_H)im][HSO_4_]	Biodiesel production from oleic acid	10.8% wt. cat., methanol:oleic acid molar ratio 6, 110 °C, 4~h	α = 92.9%, 8 cycles	[105]
SiO_2_⋅Fe_3_O_4_ [tesp(Ph-SO_3_H)_3_P][Cl] ^j^	Biodiesel production from oleic acid	0.06 g cat., benzaldehyde 30 mmol, ethylene glycol 90 mmol, cyclohexane 185 mmol, reflux, 2 h	Yields for: benzaldehyde 97% (5 cycles), propionaldehyde 96%, butanone 95%, cyclohexanone 94%	[106]
SiO_2_⋅Fe_3_O_4_ [Cl][diammonium] [HSO_4_] ^k^	Acetalization of aldehyde or ketone with ethylene glycol	0.048 cat., dimedone 1 mmol, benzaldehyde 1 mmol, 6-amino-1,3-dimethyluracil 1 mmol, 120 °C, 15–30 min	Yields for various benzaldehydes with R-group: H 94%, 3-Br 92%, 4-Br 90%, 2-Cl 88%, 4-Cl 96%, 4-Me 93%, 4-OMe 94%, 4-OH 81%	[107]
SiO_2_⋅Fe_3_O_4_ [tesp2pyr][HSO_4_] ^l^	Synthesis of pyrimido[4,5-b]quinolines.	200 mg cat., aromatic amine 1 mmol, NaNO_2_ 2.5 mmol, NaI 2.5 mmol, rt, 12–15 min	Yields for aromatic amines: C_6_H_5_NH_2_ 73%, 4-H_2_NC_6_H_4_COOH 95%, 4-NO_2_C_6_H_4_NH_2_ 83%, 4-BrC_6_H_4_NH_2_ 78%, 4-ClC_6_H_4_NH_2_ 82%, 4-MeC_6_H_4_NH_2_ 62%	[108]
SiO_2_⋅CoFe_2_O_4_ [p(b-SO_3_H)im] [OTf] ^m^	Diazotization–iodination of the aromatic amines	1:30 equimolar amount of oleic acid and the catalyst, alcohol 17.02 g, 100 °C, 4 h	CH_3_: α = 75%, C_4_H_9_: α = 40%, C_6_H_13_:α = 20%, C_8_H_17_:α = 16%	[109]
SiO_2_⋅Fe_3_O_4_ [tmsptetrazole-SO_3_H][HSO_4_] ^n^	Esterification of oleic acid with straight-chain alcohols	20 mg cat., benzaldehyde 1 mmol, 2-thiobarbituric acid 2 mmol, acetate ammonium 1 mmol, H_2_O 5 mL, rt, 35–60 min	Yields for benzaldehydes with R-groups: H 89%, 4-Cl 91%, 4-NO_2_ 95%, 4-Me 87%, 4-OMe 84%, 2-NO_2_ 93%, 2-OH 82%, 2-OMe 85%, 2–80%, 3-OMe 90%	[110]
SiO_2_⋅Fe_3_O_4_ [OH-etNH_3_][b-SO_3_] ^o^	Synthesis of pyrimidine derivatives	Aldehyde:malononitrile: thiophenol:catalyst (1/2/1/0.012; n/n/n/n), 50 °C, 5–20 min	Y = 81–91%; 5 cycles (benzaldehyde, malononitrile and thiophenol)	[111]
SiO_2_⋅Fe_3_O_4_ [tmspdabco][Cl] ^p^	Synthesis of 2-amino-3,5-dicarbonitrile-6-thio-pyridines	Aldehyde, ethyl cyanoacetate, H_2_O-polyethylene glycol	8 cycles, high yields	[112]
SiO_2_⋅Fe_3_O_4_ [tespmim][Cl] ^r^	Knoevenagel condensation	0.0007 g cat., aromatic aldehyde 1 mmol, anilines 1 mmol, thioglycolic acid 1 mmol, 70 °C, 55–70 min	(a) aniline + aromatic aldehydes Yields for R-groups in aldehydes: H 94% (10 cycles), 4-Me 88%, 4-Cl 95%, 4-NO_2_ 92%, 3-NO_2_ 89% (b) p-methylaniline + aromatic aldehydes Yields for R-groups in aldehydes: H 90%, Me 93%, 90%	[113]
SiO_2_⋅Fe_3_O_4_ [tespmim][Cl]	Synthesis of 1,3-thiazolidin-4-ones	20% mol cat., 6-amino-N,N-dimethyuracil 1 mmol, 3-(2- methyl-1H-indol-3-yl)-3-oxopropanenitrile 1 mmol, arylaldehydes 1 mmol, DMF 10 mL, 120 °C, 55–120 min	Yields for aldehydes with Ar-groups: 4-FC_6_H_4_ 90% (3 cycles), 4-ClC_6_H_4_ 90%, 4-BrC_6_H_4_ 85%, 4-CNC_6_H_4_ 90%, 4-CF_3_C_6_H_4_ 90%, C_6_H_5_ 80%, 3-ClC_6_H_4_ 90%, 3-OMeC_6_H_4_ 75%	[114]
SiO_2_⋅Fe_3_O_4_ [tespmim][Cl]	Synthesis of indole-substituted pyrido[2,3-d]pyrimidines	1% mol cat., epoxide 10 mmol, P_CO2_ = 1 Mpa, 140 °C, 4–12 h	Styrene oxide Y = 93% (11 cycles), propylene oxide Y = 99%, epichlorohydrin Y= 99%	[115]
SiO_2_⋅Fe_3_O_4_ [tespmim][Cl]	Cycloaddition of CO_2_ to epoxides	0.05 g cat., aromatic aldehyde 2 mmol, ethyl acetoacetate 2 mmol, urea/thiourea 3 mmol, 100 °C, 25–40min	Yields for aldehydes: (a) urea: Ph 95%, 3-ClC_6_H_4_ 97%, 3-NO_2_C_6_H_4_ 97%, 2-tiophen 98%, 3-FC_6_H_4_ 92% (b) thiourea: Ph 96%, 4-OMeC_6_H_4_ 90%, 2-tiophen 95%	[116]
SiO_2_⋅Fe_3_O_4_ [tespmim][Cl]	Synthesis of 3,4-dihydropyrimidin-2(1H)-ones/thiones	7 mg cat., aniline 1 mmol), formic acid 3 mmol, rt, 5–10 min	Yields for anilines with R-groups: H 99% (5 cycles), 4-Me 98%, 4-OMe 98%, 4-Cl-90%, 4-NO_2_ 98%	[117]
SiO_2_⋅Fe_3_O_4_ [tesptriazinium][Cl] ^s^	N-formylation	0.02 g cat., aromatic aldehyde 1 mmol, malononitrile 1 mmol, 5-hydroxy-2-hydroxymethyl-4H-pyran-4-one (kojic acid) 1 mmol H_2_O 5 mL, reflux, 30–45 min	Yields for benzaldehydes with R-groups: H 94%, 2,3-Cl_2_ 94%, 2,6-Cl_2_ 97%, 4-NO_2_ 98%, 3-NO_2_ 97%, 4-OH 85%	[118]
SiO_2_⋅Fe_3_O_4_ [tesampmim][Cl] ^t^	of amines	10 mg cat., benzylalcohol 1 mmol, anhydride 2 mmol, rt, 20–60 min	Yields for various benzylalcoholes with R-groups: 4-Br 96% (9 cycles), 4-OMe 94%, 4-F 94%, i-C_3_H_7_ 93%	[119]
SiO_2_⋅Fe_3_O_4_ [tmsp(alanine)im][Cl] ^u^	Synthesis of 4H-dihydropyrano	0.001 g cat., arylaldehyde 2.5 mol, arylamine 2.5 mol cyclohexanon 3 mol, EtOH 20 mL, sonication (70 W)	Yields and selectivity (anti:syn) for anilinę+ benzaldehydes with R-groups: H 92%, 99:1; 2-Cl 91%, 97:3; 4-Me 88%, 99:1; 4-Cl 92%, 99:1; 4-Br 92%, 99:1; 4-OMe 89%, 99:1; 2-OMe 86%, 99:1	[120]
SiO_2_⋅Fe_3_O_4_ [tespdeaim][PF_6_] ^w^	[3,2-b]pyran- 3-carbonitrile	25 mg cat., aldehyde or ketone 2 mmol, malonitrile 2 mmol, water 10 mL, 30 °C, 1 h	α for aldehydes/ketones: cyclohexanone >99%, furfural >99%, benzaldehyde >99%, 4-nitrobenzaldehyde 91.6%, 4-hydroxybenzaldehyde 89.4%, 2-hydroxybenzaldehyde 80.3%, 2-methylpropanal 92%	[121]
Fe_3-x_Ti_x_O_4_-SiO_2_ [TrpEt_3_][I] ^x^	Derivatives	0.12 g cat., anilines 1 mmol, dialkyl acetylenedicarboxylates 1 mmol, terminal alkynes or acetophenones 1.2 mmol, 100 °C, 15–18 h	Methyl 4-propylquinoline2-carboxylate: Y = 75% ethyl 6-hydroxy4-propylquinoline-2-carboxylate: Y = 92%	[122]
Fe_3-x_Ti_x_O_4_-SiO_2_ [TrpEt_3_][I]	Acetylation of alcohols	0.12 g cat., anilines 1 mmol, dialkyl acetylenedicarboxylates 2.2 mmol, 100 °C, 10–22 h	Ethyl 4-(4-bromophenyl)benzo quinoline-2-carboxylate: Y = 77% dimethyl 8-nitroquinoline2,4-dicarboxylate: Y = 82%	[122]

^a^ 1-methyl-3-(trimethoxysilylpropyl)imidazolium chloride—chloroaluminate (III), ^b^ *N*-(trimethoxysilylpropyl)imidazolium chloride—chlorozincate (II), ^c^ 1-methyl-3-(trimethoxysilylpropyl)imidazolium hydrogensulfate, ^d^ 1-methyl-3-(trimethoxysilylpropyl)imidazolium hydrogensulfate, ^5^*N*-(trimethoxysilylpropyl)-5-phenyl-1H-tetrazolium-SO_3_H chloride, ^e^ *N*-(3-sulfopropyl)-*N*-(3-propyltrimethoxysilane)triethylenediammonium ditriflate, ^f^ 1-methyl-3-(trimethoxysilylpropyl)imidazolium dihydrogenphosphotungstate, ^g^ 3-(4-sulfobutyl)-1-(3-propyltriethoxysilane)imidazolium hydrogensulfate, ^h^ 3-(3-sulfopropyl)-1-(3-propyltriethoxysilane)imidazolium hydrogensulfate, ^i^ *P*-(trimethoxysilylpropyl)-*P*,*P*,*P*-tri(4-sulfophenyl)phosphonium chloride, ^j^ *N*-(trimethoxysilylpropyl)-*N,N*-dimethyl-*N*-(dimethylammonium)ammonium chloride hydrosulfate, ^k^ *N*-(propyl-triethoxysilane)-2-pyrrolidinium hydrogensulfate, ^l^ 3-(4-sulfobutyl)-1-(3-trimethoxysilylmerkaptopropyl)imidazolium triflate, ^m^ *N*-(trimethoxysilylpropyl)-5-phenyl-1H-tetrazolium-sulfobutyl hydrogensulfate, ^n^ 2-hydroxyethylammonium butylsulphonate, ^o^ *N*-(3-propyltrimethoxysilane)triethylenediammonium chloride, ^p^ 1-methyl-3-(triethoxysilylpropyl)imidazolium chloride, ^r^ N-(triethoxysilylpropyl)triazinium chloride, ^s^ 3-((3-(trisilyloxy)propyl)propionamide)-1-methylimidazolium chloride, ^t^ 3-(trimethoxysilylpropyl)-1-(2-aminopropanoate)imidazolium trimethylethanolammonium chloride, ^u^ imidazolium alanine based IL, ^w^ 3-(trimethoxysilylpropyl)-1-(triethylamine)imidazolium hexafluorophosphate, ^x^ triethyltryptophanium iodide.

**Table 4:** Examples of silica-based SILLP as a matrix or co-catalyst in organic catalysis.

Catalyst	Reaction Type	Reaction Conditions	Reaction Parameters	Lit.
SiO_2_/Rh [tespbim][BF_4_] ^a^/(tppti) ^b^	Hydroformylation of 1-hexene	CO/H_2_(1:1; *v*/*v*), Rh/P (1:10, n/n), 100 °C, 5 h	α = 33%, S = 2.4 (n/i-heptanal ratio), TOF = 65 min^−1^	[124,125,126]
SiO_2_/Ni [tesp(p-SO_3_H)im][OTf] ^c^	Hydrogenation of n-valeraldehyde	4.5 g cat., n-valeraldehyde 30 mL, P_H2_ = 3 MPa, 200 °C 8 h	α = 100%, S = 98.6%	[127]
SiO_2_/PbS [tespmim][Cl] ^d^	Dehydrogenation of formic acid	0.0007 g cat., HCOOH/HCOONa 9.00 mmol, 8:1; n/n, H_2_O 2.5 mL, 40 °C, 750 rpm	Y = 97% (formic acid decomposition), S_H2_ = 78%, TOF = 604 h^−1^	[128]
SiO_2_/Pd [bvim][Br] ^e^	Suzuki coupling	1% mol. cat. phenylboronic acid:aryl halide (1.1:1; n/n), H2O/EtOH (1.2 mL; 1:1; *v*/*v*), K_2_CO_3_ (0.6 mol), 50 °C, 19 h	Yields for aryl bromides with R-groups: 4-CHO 81%, 4-OMe 89%, 3-OMe 85%, 4-NO_2_ 80%, 2-CHO 95%, 4-COCH_3_ 88%, 3-COCH_3_ 70%, 4-COOH 88%, 2-CH_3_ 86%, 2-CN 88%, 3,5-(CF_3_)_2_ 89%, H 78%, 1-naphthyl 85%)	[129]
SiO_2_/POSS ^f^/Pd [tesppim][Cl] ^g^/[tespmim][Cl]	Suzuki coupling	0.07% mol. cat. phenylboronic acid:aryl halide (1.1:1; n/n), H2O/EtOH (1.2 mL; 1:1; *v*/*v*), K_2_CO_3_ (0.6 mol), 50 °C, 19 h	Yields and TOF for aryl bromides with R-groups: 4-CHO 99%, 1429 h^−1^;4-OMe 95%, 1327 h^−1^; 3-OMe 75%, 1071 h^−1^; 4-NO_2_ 99%, 1429 h^−1^; 4-COCH_3_ 99%, 1429 h^−1^; 3-COCH_3_ 99%,1429 h^−1^; 3-CH_3_ 99%, 1414 h^−1^; 4-CH_3_ 93%, 1329 h^−1^; 4-CN 99%, 1429 h^−1^	[130]
SiO_2_/POSS/Pd [tesppim][Cl]/[tespmim][Cl]	Heck reaction	0.07% mol. cat. aryl halide, 0.5 mmol, methyl acrylate 0.75 mmol, triethylamine 1 mmol, DMF 1 mL, 120 °C, 3 h	Yields and TOF for aryl iodides with R-groups: H >99%, 476 h^−1^; 4-CH_3_ >99%, 476 h^−1^; 4-COCH_3_ 99%, 471 h^−1^; 4-OCH_3_ 99%, 471 h^−1^; 3-OCH_3_ 99%, 471 h^−1^; 4-NO_2_ >99%, 476 h^−1^; 2-C_4_H_3_S 91%, 433 h^−1^; 4-CHO >99%, 286 h^−1^	[130]
SiO_2_/Pd [bvim][Br]	Suzuki coupling	0.1% mol. cat. phenylboronic acid:aryl halide (45.2:40; n/n mmol), 0.33 M EtOH (121.2 mL), K_2_CO_3_ (48 mmol), 50 °C, 1.5 mLmin^−1^, 36 h	Yields for different aryl bromides (H 96%, CH_3_ 96%, CHO 98%) TON = 3800	[131]
SiO_2_/Proline [bvim][NTf_2_] ^h^	Asymmetric aldol reaction	5% mol cat., aldehyde 1 mmol, cyclohexanone 5 mmol, 1.2 mmol H_2_O, rt, 2.5 h	Yields and enantiomeric excess (ee) for aldehydes: 4-NO_2_Ph Y = 99%, ee = 98%; 4-ClPh Y = 92%, ee = 99%; 4-BrPh Y = 95%, ee = 97%, 4-CNPh Y = 99%, ee = 92%	[132]
SiO_2_/CALB ^i^ [tespmim][BF_4_] ^j^	Diacylglycerol production	5% wt. cat., corn oil 4.4 g, glycerol 0.23 g, tert-pentanol 17 mL, 50 °C, 12 h	α = 70.94%, 5 cycles	[133]
SiO_2_/PPL ^k^ [tmspmim][BF_4_] ^l^	Triacetin hydrolysis	6.83 g of glyceryl triacetate, pH = 7, 45 °C, 10 min	5 cycles	[134]
SiO_2_⋅Fe_3_O_4_/CRL ^m^ [tespmim][Cl]	Production of trans-free plastic fats	Palm stearin or liquid rice bran oil, 45 °C, 48 h	4 cycles	[135]

^a^ 1-butyl-3-(triethoxysilylpropyl)imidazolium tetrafluoroborate, ^b^ tri(m-sulfonyl)triphenyl phosphine tris(1-butyl-3-methyl-imidazolium) salt as a ligand, ^c^ 3-(3-sulfopropyl)-1-(3-propyltriethoxysilane)imidazolium triflate, ^d^ 1-methyl-3-(triethoxysilylpropyl)imidazolium chloride, ^e^ 1,4-bis(3-vinylimidazolium-1-yl) bromide, ^f^ polyhedral oligomeric silsesquioxanes, ^g^ 1propyl-3-(triethoxysilylpropyl)imidazolium chloride, ^h^ 1,4-bis(3-vinylimidazolium-1-yl) bis(trifluoromethane)sulfonimide, ^i^ *Candida antarctica* lipase B, ^j^ 1-methyl-3-(triethoxysilylpropyl)imidazolium tetrafluoroborate, ^k^ *Porcine pancreas* lipase, ^l^ 1-methyl-3-(trimethoxysilylpropyl)imidazolium tetrafluoroborate, ^m^ *Candida rugosa* lipase.

## Data Availability

Data sharing is not applicable for this article.
